# Ebola virus triggers receptor tyrosine kinase-dependent signaling to promote the delivery of viral particles to entry-conducive intracellular compartments

**DOI:** 10.1371/journal.ppat.1009275

**Published:** 2021-01-29

**Authors:** Corina M. Stewart, Alexandra Phan, Yuxia Bo, Nicholas D. LeBlond, Tyler K. T. Smith, Geneviève Laroche, Patrick M. Giguère, Morgan D. Fullerton, Martin Pelchat, Darwyn Kobasa, Marceline Côté

**Affiliations:** 1 Department of Biochemistry, Microbiology and Immunology, University of Ottawa, Ottawa, Canada; 2 Ottawa Institute of Systems Biology, University of Ottawa, Ottawa, Canada; 3 Centre for Infection, Immunity, and Inflammation, University of Ottawa, Ottawa, Canada; 4 Centre for Catalysis Research and Innovation, University of Ottawa, Ottawa, Canada; 5 Special Pathogens Program, National Microbiology Laboratory, Public Health Agency of Canada, Winnipeg, Canada; 6 Department of Medical Microbiology, University of Manitoba, Winnipeg, Canada; Division of Clinical Research, UNITED STATES

## Abstract

Filoviruses, such as the Ebola virus (EBOV) and Marburg virus (MARV), are causative agents of sporadic outbreaks of hemorrhagic fevers in humans. To infect cells, filoviruses are internalized via macropinocytosis and traffic through the endosomal pathway where host cathepsin-dependent cleavage of the viral glycoproteins occurs. Subsequently, the cleaved viral glycoprotein interacts with the late endosome/lysosome resident host protein, Niemann-Pick C1 (NPC1). This interaction is hypothesized to trigger viral and host membrane fusion, which results in the delivery of the viral genome into the cytoplasm and subsequent initiation of replication. Some studies suggest that EBOV viral particles activate signaling cascades and host-trafficking factors to promote their localization with host factors that are essential for entry. However, the mechanism through which these activating signals are initiated remains unknown. By screening a kinase inhibitor library, we found that receptor tyrosine kinase inhibitors potently block EBOV and MARV GP-dependent viral entry. Inhibitors of epidermal growth factor receptor (EGFR), tyrosine protein kinase Met (c-Met), and the insulin receptor (InsR)/insulin like growth factor 1 receptor (IGF1R) blocked filoviral GP-mediated entry and prevented growth of replicative EBOV in Vero cells. Furthermore, inhibitors of c-Met and InsR/IGF1R also blocked viral entry in macrophages, the primary targets of EBOV infection. Interestingly, while the c-Met and InsR/IGF1R inhibitors interfered with EBOV trafficking to NPC1, virus delivery to the receptor was not impaired in the presence of the EGFR inhibitor. Instead, we observed that the NPC1 positive compartments were phenotypically altered and rendered incompetent to permit viral entry. Despite their different mechanisms of action, all three RTK inhibitors tested inhibited virus-induced Akt activation, providing a possible explanation for how EBOV may activate signaling pathways during entry. In sum, these studies strongly suggest that receptor tyrosine kinases initiate signaling cascades essential for efficient post-internalization entry steps.

## Author summary

Ebola virus (EBOV) and Marburg virus (MARV) are zoonotic pathogens that can cause severe hemorrhagic fevers in humans and non-human primates. They are members of the growing *Filoviridae* family that also includes three other species of Ebolaviruses known to be highly pathogenic in humans. While vaccines for EBOV are being deployed and showed high efficacy, pan-filoviral treatment is still lacking. To infect cells, EBOV requires the endosomal/lysosomal resident protein Niemann-Pick C1 (NPC1). Accordingly, viral particles require extensive trafficking within endosomal pathways for entry and delivery of the viral genome into the host cell cytoplasm. Here, we used chemical biology to reveal that EBOV triggers receptor tyrosine kinase (RTK)-dependent signaling to traffic to intracellular vesicles that contain the receptor and are conducive to entry. The characterization of host trafficking factors and signaling pathways that are potentially triggered by the virus are important as these could be targeted for antiviral therapies. In our study, we identified several RTK inhibitors, some of which are FDA-approved drugs, that potently block EBOV infection. Since all filoviruses known to date, even Měnglà virus that was recently discovered in bats in China, use NPC1 as their entry receptor, these inhibitors have the potential to be effective pan-filovirus antivirals.

## Introduction

In order for viruses to replicate, they must first deliver their genetic material into host cells. With regards to enveloped viruses specifically, this requires fusion of the viral membrane with the cellular membrane, a process that is mediated by viral fusion proteins that protrude from the viral envelope. Upon specific triggers, including but not limited to viral receptor interaction, these viral fusion proteins will undergo extensive conformational rearrangements to facilitate membrane fusion [1]. In the last few years, some enveloped viruses, such as Ebola virus (EBOV) and Lassa fever virus (LFV), have been found to require interaction with entry receptors localized in late endosomes and/or lysosomes [2–5]. In these instances, the viral particles must not only be endocytosed, but also require trafficking to the specific intracellular compartment containing host factors necessary for fusion triggering. However, it is still unclear whether these viruses use mechanisms to regulate their trafficking within the endosomal system or are passive passengers of intracellular vesicles.

Filoviruses, including EBOV and Marburg virus (MARV), are zoonotic pathogens that can cause severe hemorrhagic fevers in humans and non-human primates [6]. Previous studies have shown that the filovirus entry receptor is the late endosome/lysosome-resident protein Niemann-Pick C1 (NPC1) [2–4]. To reach NPC1, filoviral particles must be internalized via macropinocytosis and then undergo extensive trafficking through the endosomal labyrinth whereby endosomes transition from Rab5+ to Rab7+ compartments [7–9]. Also involved in filovirus trafficking to NPC1 are the homotypic fusion and protein sorting (HOPS) and PIKfyve/ArPIKfyve/Sac3 (PAS) tethering and trafficking complexes [2,10–12]. These complexes, along with the small GTPase Rab7, regulate vesicular fusion events required for specific trafficking of cargoes to late endosomes and lysosomes in cells [13,14]. While the activity of Rab7, HOPS, and PAS can be modulated by various stimuli [15,16], whether EBOV can also regulate these host trafficking proteins remains to be determined.

Using a probe that binds to phosphatidylinositol (3,5)bisphosphate (PtdIns(3,5)P2), the product of PIKfyve within the PAS complex, we previously found that PtdIns(3,5)P2 was increased during EBOV entry, suggesting that viral particles can stimulate the activity of the PAS complex [10]. Although the mechanism through which this occurs is still unknown, previous studies have shown that Akt, part of the phosphoinositide 3-kinase (PI3K)/Akt pathway, can activate PIKfyve [16]. Furthermore, *Saeed et al*. found that EBOV triggers Akt phosphorylation and that the PI3K/Akt pathway was required for EBOV trafficking [17]. Therefore, it is plausible that EBOV stimulates the activity of the PAS complex through activating the PI3K/Akt signaling pathway. However, the mechanism through which EBOV may activate Akt, a primarily cytosolic protein, is still unclear. We propose that EBOV activates host signaling pathways upon contact with the host cell that function to “prime” the cell for both entry and subsequent replication.

To identify signaling pathways important for filovirus entry, we screened a library of kinase inhibitors using pseudotypes harboring the EBOV, MARV or vesicular stomatitis virus (VSV) glycoproteins. We found that receptor tyrosine kinase (RTK) inhibitors potently block EBOV and MARV GP-dependent viral entry. More specifically, inhibitors of epidermal growth factor receptor (EGFR), c-Met, and the insulin receptor (InsR)/insulin like growth factor 1 receptor (IGF1R) blocked filoviral GP-mediated entry and growth of replicative EBOV. Interestingly, while the c-Met and InsR/IGFR inhibitors both interfered with EBOV trafficking to NPC1, virus delivery to the receptor was not impaired in the presence of an EGFR inhibitor. However, the NPC1+ compartments were altered phenotypically and were rendered incompetent to permit viral entry. Interestingly, despite their different mechanisms of action, all three RTK inhibitors tested inhibited virus-induced Akt activation. These studies strongly suggest that receptor tyrosine kinases initiate signaling cascades that are essential for efficient EBOV post-internalization entry steps.

## Results

### Screening of a kinase inhibitor library for the identification of signaling pathways required for filovirus GP-mediated entry

To identify signaling pathways important for filovirus entry, we screened a library of small-molecule kinase inhibitors using murine leukemia virus (MLV) pseudotyped with the glycoproteins of EBOV, MARV, or VSV. Due to the toxicity inherent to the mucin domain of EBOV GP that leads to low virus yield, we used an EBOV GP construct with this region deleted (EBOV GPΔmuc, herein simply referred to as EBOV GP)[18]. Previous work has shown that this construct does not interfere with the overall EBOV entry pathway and likewise requires cathepsin cleavage and NPC1 binding to mediate infection [19]. In addition, to minimize the effect of signaling induced by growth factors found in the serum used to culture the cells, Vero cells were serum-starved during pre-incubation with 1μM of each inhibitor. MLV pseudotypes encoding β-galactosidase (LacZ) were then added for four hours, after which cells were placed in media containing ammonium chloride to stop entry. Twenty-four hours post-infection, media was replaced again, and cells incubated for another 48 hours to allow for the expression of the reporter gene. Transduction efficiency was measured using a luminescence-based substrate and normalized to vehicle-treated cells. Ratios of signals from EBOV or MARV pseudotypes over that of VSV were then calculated. VSV pseudotypes were used as controls because the entry pathway of VSV is different than that of filoviruses; it is internalized via clathrin-dependent endocytosis and VSV G-mediated fusion occurs in early endosomes [20,21]. In addition, inhibitors that exert effects on MLV reverse-transcription, integration, or reporter gene transcription are expected to have similar effects on all pseudotypes, including VSV, and as such will not be identified as hits in our analysis. We also assessed potential effects of inhibitor treatment on cellular proliferation and metabolic activity using CellTiter Glo 24 hours post-treatment. Transduction data from inhibitors that had metabolic activity lower than 80% of the vehicle controls were eliminated.

**Fig 1:**
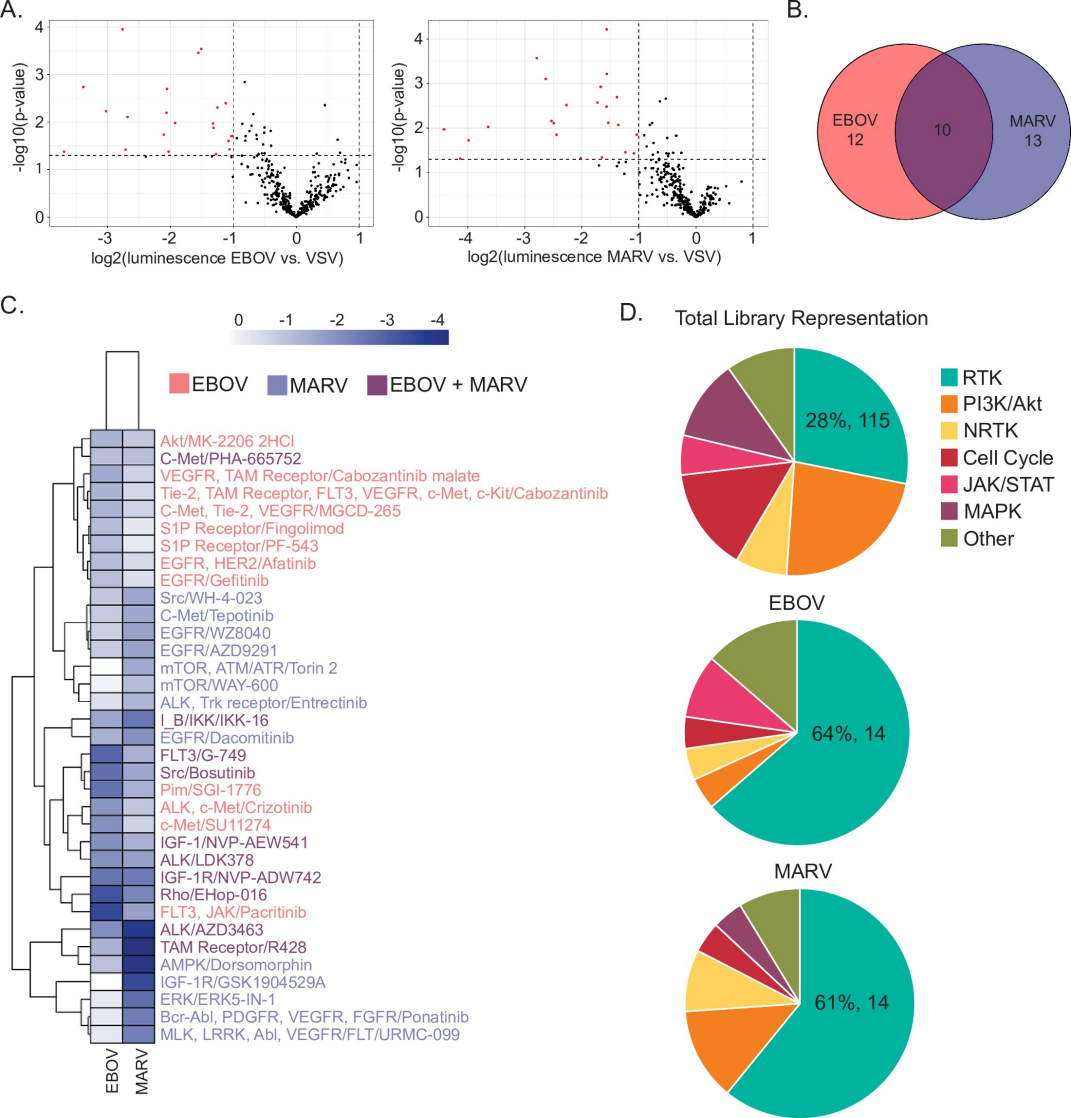
A kinase inhibitor screen to identify signaling pathways required for EBOV and MARV GP-mediated entry. MLV pseudotypes encoding LacZ and harboring EBOV, MARV, or VSV glycoproteins were used to screen the Selleckchem L1200 kinase inhibitor library at 1μM in Vero cells (A) Volcano plots of the log2 values of the ratios of the means of biological replicates of EBOV (left panel) or MARV (right panel) vs VSV pseudotype transduction relative to the DMSO controls over the–log10 values of the p-value. Dots in red represent hits as determined with at least 2-fold ratio and p<0.05. Results are means of three independent experiments performed in duplicates. (B) Venn diagram of the hits for EBOV and MARV, with 22 total hits for EBOV, 23 total hits for MARV, and 10 hits shared between the two viruses. (C) Heat maps of the log2(normalized transduction efficiency of EBOV or MARV vs. that of VSV) for the total hits for both viruses. (D) Pie charts of the signaling pathways (grouped into the following categories: receptor tyrosine kinase (RTK) inhibitors, Janus kinase/signal transducers and activators of transcription (JAK/STAT) pathway inhibitors, mitogen-activated protein kinase (MAPK) pathway inhibitors, non-receptor tyrosine kinase (NRTK) inhibitors, and phosphatidylinositol-3-kinase (PI3K)/Akt inhibitors, or others) of the inhibitors in the library (top), EBOV hits (middle), and MARV (bottom). The representation in percentage and the number of the RTK inhibitors is indicated.

Volcano plots of the p-values versus ratios over VSV revealed 35 compounds that had a significant effect on EBOV and/or MARV GP-mediated infection when compared to VSV (Fig 1A and 1B, S1 Data, S1 and S2 Tables). Interestingly, none of the 35 compounds enhanced infection–all were found to be inhibitory. Of the 35 hits, 10 were shared by EBOV and MARV, while 12 and 13 were unique to EBOV or MARV GP-mediated infection respectively (Fig 1B), suggesting some overlap between the signaling pathways used by these filoviruses. Importantly, some hits and their targets were previously shown to have an effect on filovirus GP-mediated infection, including AMP-activated protein kinase (AMPK) (Dorsomorphin)[22] and Akt (MK-2206) [17,22], thus validating our approach (Fig 1C). Some new pathways were also revealed, such as mammalian target of rapamycin (mTOR), mitogen-activated protein kinase (MAPK), and sphingosine-1-phosphate receptors (S1PRs) (Fig 1C and 1D). Interestingly, we also observed an enrichment of receptor tyrosine kinase (RTK) inhibitors in our hits compared to the overall proportion of RTK inhibitors in the library (Fig 1D), suggesting that RTK signaling is important for EBOV and MARV entry. In sum, the results of this small molecule screen indicate that specific signaling pathways, particularly RTK signaling, are required for filovirus entry.

### Receptor tyrosine kinase inhibitors block entry of multiple filoviruses and growth of replicative EBOV in Vero cells

**Fig 2:**
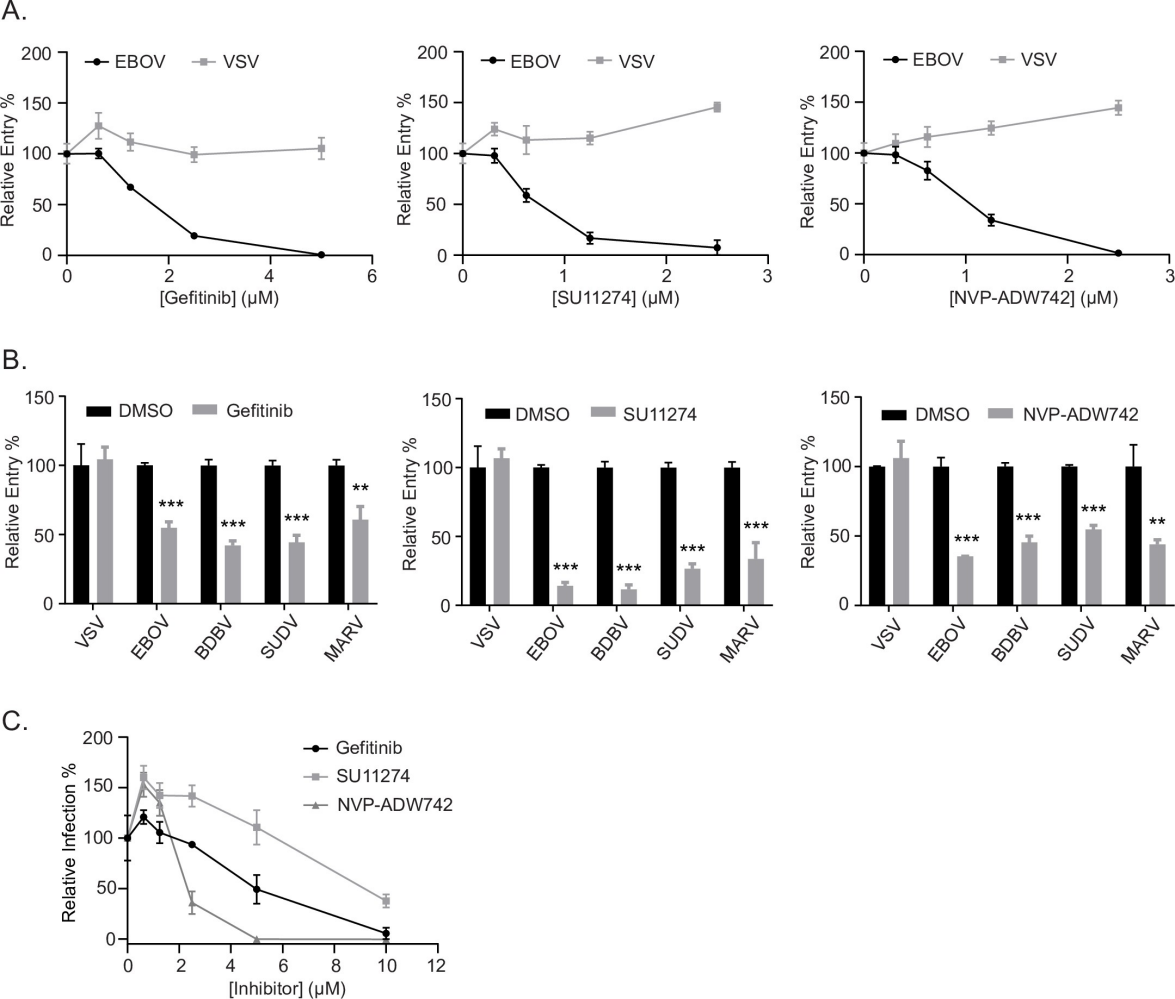
RTK inhibitors block filovirus entry in Vero cells. (A) Entry of βlam VLPs harboring the EBOV GP or VSV G in Vero cells treated with vehicle (DMSO, 0.1%) or increasing concentrations of Gefitinib, SU11274, or NVP-ADW742. Entry was detected via flow cytometry after loading cells with βlam substrate (CCF2) and measuring the percentage of inhibitor treated cells with cleaved CCF2. Data are expressed as percentages relative to DMSO-treated cells. (B) Entry of βlam VLPs bearing the GPs of EBOV, SUDV, BDBV, MARV, or VSV G in the presence of 1 μM Gefitinib, 1 μM SU11274, 1 μM NVP-ADW742, or vehicle (DMSO, 0.1%). (C) Infection of Vero cells with replication-competent EBOV expressing GFP at increasing concentrations of the indicated inhibitor. Infection was measured by GFP fluorescence 3 days post-infection and normalized to vehicle-treated cells. Results are expressed as mean ± s.d. of triplicates and are representative of 3 experiments. * p < 0.05, ** p < 0.01, *** p < 0.001.

To confirm the effect of RTK inhibitors on EBOV GP-mediated entry, we used filoviral-like particles (VLPs) generated by co-expression of the EBOV nucleoprotein (NP), matrix protein (VP40), and the glycoprotein of interest [23]. These viral particles exhibit the characteristic filamentous morphology of filoviral particles, can enter target cells according to the entry pathway of the viral glycoproteins expressed on their surface, and allow measurement of membrane fusion in cells by using a VP40 construct that is fused with β-lactamase (βlam) [23]. Using this system, we first tested a panel of RTK inhibitors identified as screen hits and measured viral entry into Vero cells using VLPs bearing EBOV GP or VSV G (S1 Fig). We found that RTK inhibitors of EGFR, c-Met, InsR/IGF1R, or multiple RTKs, blocked EBOV GP-mediated entry and had no significant effect on VSV G-mediated entry (S1 Fig). To further investigate and dissect the RTK signaling requirements, we performed dose-response studies of the specific inhibitors of EGFR (Gefitinib), c-Met (SU11274), or InsR/IGF1R (NVP-ADW742) and found that all reduced EBOV GP-mediated entry (Gefitinib IC50 = 1.8 μM, SU11274 IC50 = 0.7 μM, NVP-ADW742 IC50 = 1 μM) (Fig 2A). Importantly, these had either no effect or slightly increased VSV G-mediated entry (Fig 2A). To confirm that the absence of the mucin domain did not influence the potency of the RTK inhibitors, we infected Vero cells with EBOV VLPs bearing EBOV ΔM GP or EBOV full length GP and did not observe a difference between the two GPs (S2 Fig). Additionally, we tested entry mediated by two other highly pathogenic ebolaviruses, Bundibugyo (BDBV) and Sudan (SUDV). We found that the three RTK inhibitors blocked entry mediated by all filovirus GPs tested in our panel, (Fig 2B), suggesting that they interfere with an entry step or steps required by all filoviruses.

To investigate the efficacy of the RTK inhibitors on native infection, we next tested their ability to block growth of GFP-expressing replicative EBOV. We found that all drugs were able to inhibit EBOV growth, albeit with different efficiency (Fig 2C). The InsR/IGF1R inhibitor, NVP-ADW742, was the most potent and completely blocked replicative EBOV infection at 5μM, while the c-Met inhibitor, SU11274, required 10μM for complete inhibition. Although viral replication was still observed in the presence of Gefitinib at 10μM, the compound was able to reduce EBOV growth (Fig 2C). Taken together, these results indicate that RTK inhibitors can block entry by several pathogenic filoviruses and growth of replicative EBOV.

### The antiviral effects of Gefitinib, SU11274, and NVP-ADW742 are specific to filoviral entry

**Fig 3:**
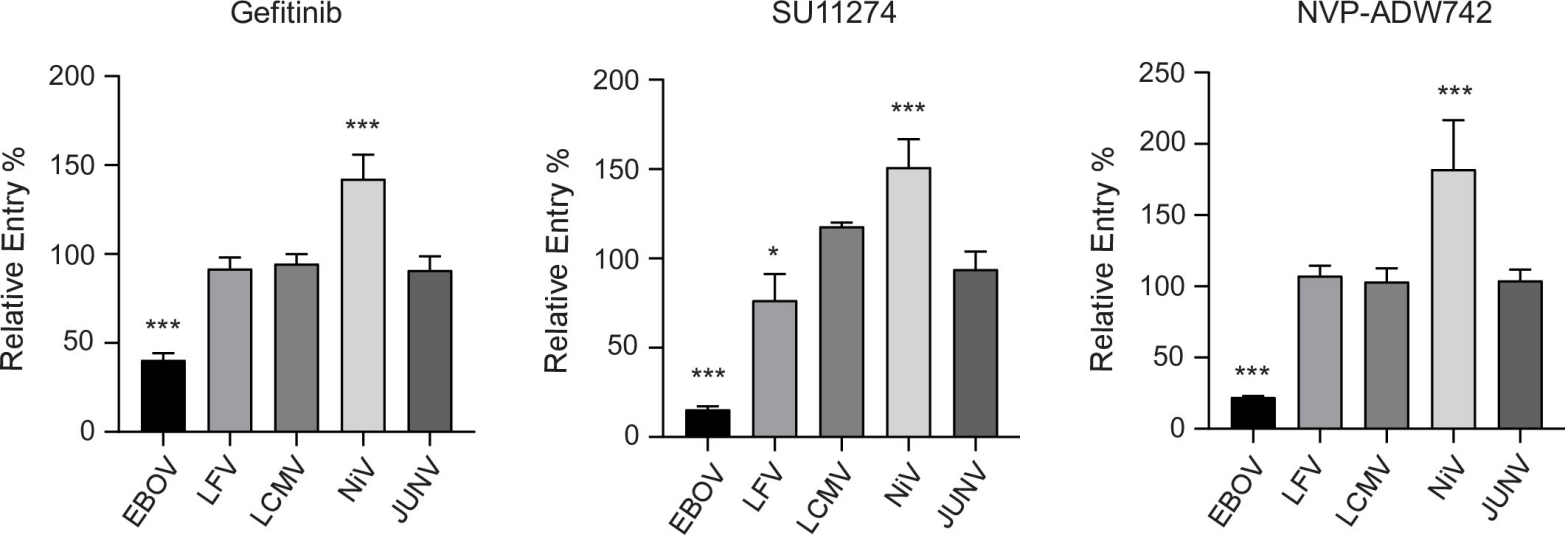
RTK inhibitors do not inhibit entry mediated by a panel of other viral GPs. Vero cells were transduced with MLV pseudotypes encoding LacZ and harboring EBOV, LFV, LCMV, Nipah, or Junin glycoproteins in the presence of Gefitinib (1 μM), SU11274 (1 μM), NVP-ADW742 (1 μM) or vehicle (DMSO, 0.1%). Results are expressed as mean ± s.d. of triplicates and are representative of 3 experiments. * p < 0.05, ** p < 0.01, *** p < 0.001.

To investigate the specificity of the RTK inhibitors on filoviral entry, we next tested entry of MLVs bearing glycoproteins from viruses that utilize a variety of different entry routes. Specifically, we used MLV pseudotypes of LFV, lymphocytic choriomeningitis virus (LCMV), and Junin virus (JUNV), which undergo fusion in late or early endosomes following internalization, in addition to Nipah virus (NiV), which utilizes the ephrin receptors for viral entry at the cell surface [24–26]. We found that entry mediated by the LCMV and JUNV glycoproteins was unchanged by the EGFR, InsR/IGF1R, and c-Met inhibitors despite their shared requirement for internalization and endosomal trafficking (Fig 3) [27,28]. LFV entry, which requires low pH and LAMP1 in late endosomes [5], remained mostly unaffected by the inhibitors, although a slight decrease was observed in cells treated with the c-Met inhibitor, SU11274 (Fig 3). This agrees with a recently published study implicating c-Met as a host factor required for LFV entry [29]. Interestingly, NiV entry, which occurs at the cell surface [24], was increased in the presence of the inhibitors (Fig 3). These results further support the notion that the RTK inhibitors block specific step(s) in filovirus entry.

### The effect of RTK inhibitors on EBOV GP-mediated entry in bone marrow-derived macrophages

**Fig 4:**
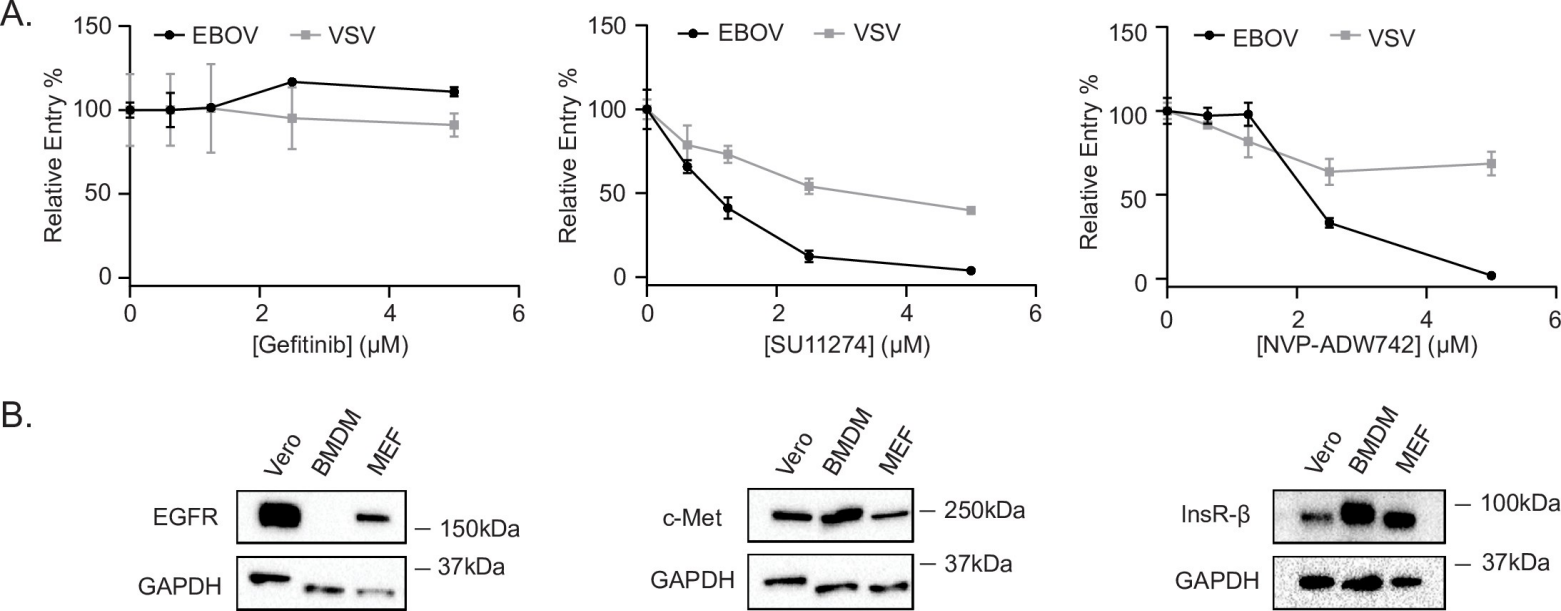
Effect of RTK inhibitor treatment on EBOV GP-mediated entry in BMDMs. (A) Entry of βlam VLPs harboring EBOV GP or VSV G in BMDMs treated with vehicle (DMSO, 0.1%) or increasing concentrations of Gefitinib, SU11274, or NVP-ADW742. (B) Vero cells, BMDMs, and MEFs were serum-starved, lysed, and expression of EGFR, Met, InsR-β, and GAPDH was detected by immunoblotting. Results in (A) are expressed as mean ± s.d. of triplicates and are representative of 3 experiments. Results in (B) are representative blots of 3 independent experiments.

Previous studies have shown that one of the primary targets of filoviruses *in vivo* are macrophages [30]. Therefore, we assessed EBOV entry in bone marrow-derived macrophages (BMDMs) in the presence of the inhibitors. We found that while both SU11274 and NVP-ADW742 efficiently blocked EBOV GP-mediated entry, Gefitinib had no effect (Fig 4A). Entry of VSV G-bearing VLPs into BMDMs was also slightly reduced in the presence of SU11274 and NVP-ADW742 (Fig 4A). The lack of inhibitory effect in BMDMs after Gefitinib treatment prompted us to investigate the expression of the inhibitors’ targets in both cell types. We found that c-Met and InsR, respective targets of SU11274 and NVP-ADW742, were expressed in both Vero cells and BMDMs (Fig 4B). However, expression of EGFR was undetectable in BMDMs (Fig 4B). Furthermore, this apparent absence of expression could not be attributed to an inability of the antibody to recognize mouse EGFR, since it could readily detect the protein in mouse embryonic fibroblasts (Fig 4B). Overall, these data suggest that RTK inhibitors can efficiently block EBOV entry in different cell types and that the effect is dependent on expression of the specific RTKs targeted by these inhibitors.

### The c-Met and InsR/IGF1R inhibitors interfere with EBOV trafficking to NPC1+ intracellular compartments

**Fig 5:**
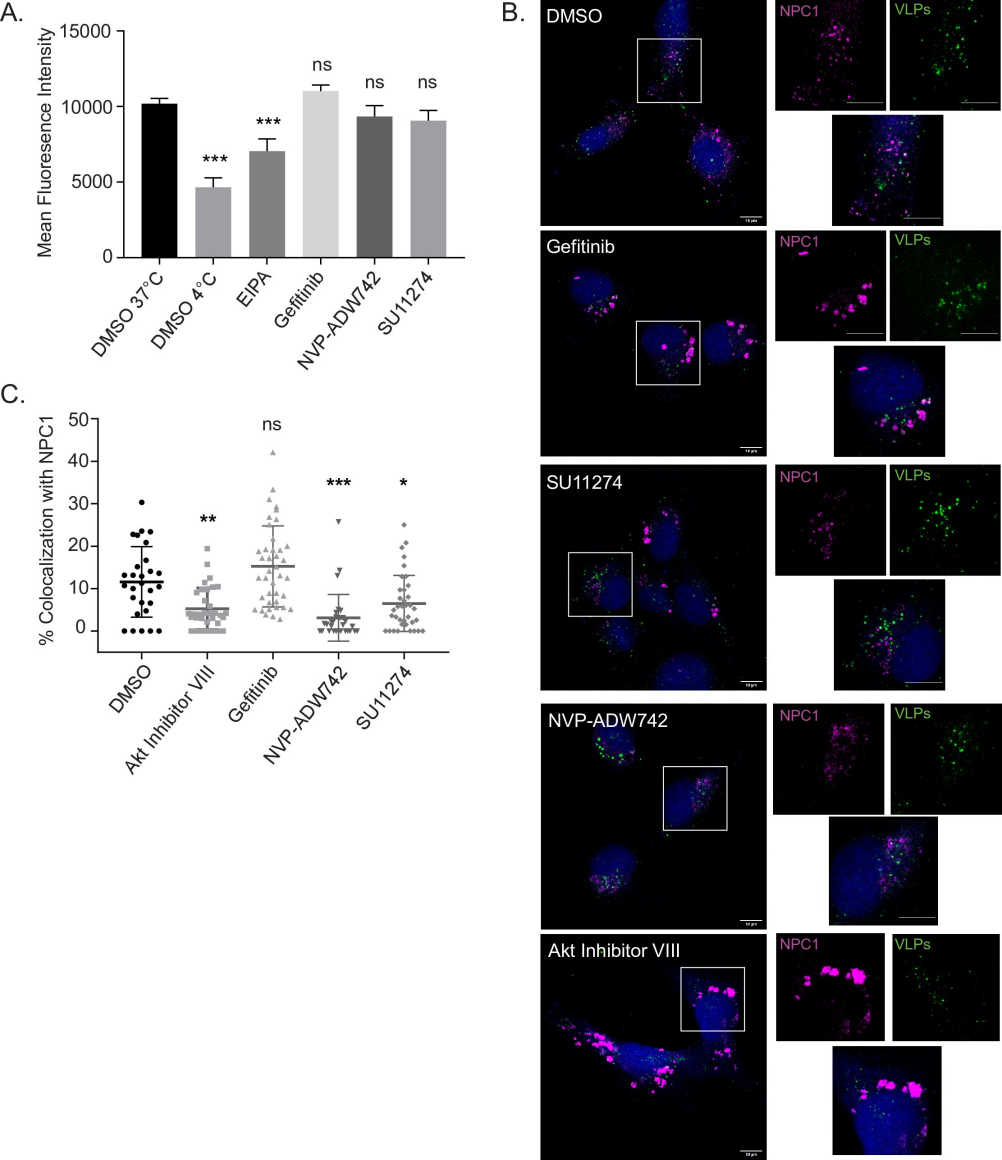
RTK inhibitors do not block EBOV VLP internalization, but SU11274 and NVP-ADW742 interfere with trafficking to NPC1+ compartments. (A) Fluorescent mCherry VLPs harboring EBOV ΔM GP were pre-bound to Vero cells by spinoculation at 4°C, followed by washing and incubation with vehicle (DMSO, 0.1%), EIPA (30 μM), Gefitinib (5 μM), SU11274 (2.5 μM), or NVP-ADW742 (2.5 μM) at 37°C for one hour, or vehicle (DMSO, 0.1%) at 4°C for one hour. Cells were then trypsinized with 0.5% trypsin and mCherry fluorescence analyzed by flow cytometry. (B) Infection of HT1080 cells pre-treated with DMSO (0.1%), Akt Inhibitor VIII (10 μM), Gefitinib (5 μM), SU11274 (2.5 μM), or NVP-ADW742 (2.5 μM) with fluorescent VLPs (Green) harboring the fusion deficient ΔM GP^F535R^ for 3 h. 30 min prior to fixation, CMAC cytoplasmic dye (Blue) was added. Cells were then fixed, permeabilized, and immunostained with rabbit anti-NPC1 and DY650-conjugated antiserum (Magenta). Cells were imaged on an LSM800 confocal microscope (Zeiss). Images are displayed as maximum intensity z-projections, bar = 10 μm. (C) Colocalization between VLPs and NPC1 (of a minimum of 25 cells per condition) were analyzed using Imaris software (Bitplane). Data are representative of 3 independent experiments. * p < 0.05, ** p < 0.01, *** p < 0.001.

Previous studies have shown that influenza A virus (IAV) uses EGFR signaling for internalization into host cells [31]. To test whether RTK signaling is also required for EBOV internalization, we used fluorescent EBOV VLPs containing VP40 fused to mCherry and spinoculated them onto Vero cells at 4°C to prevent internalization. Unbound VLPs were removed, pre-warmed media containing inhibitors or vehicle was added, and cells were incubated at 37°C for 1 hour to allow for VLP internalization. VLPs that were not internalized were then removed from the cell surface using trypsin and the level of fluorescent VLP internalization was measured by flow cytometry. For these experiments, we used the macropinocytosis inhibitor 5-(N-Ethyl-N-isopropyl)amiloride (EIPA) as a control [32]. We found that while EIPA reduced VLP internalization in cells, IC90 concentrations of Gefitinib, SU11274, or NVP-ADW742 did not interfere with EBOV internalization (Fig 5A). This indicates that, unlike IAV, EBOV requires RTK signaling for a post-internalization step or steps.

To further investigate the mechanism by which RTK inhibitors block EBOV GP-mediated entry, we used fluorescent VLPs harboring the fusion deficient EBOV GP^F535R^ [33]. VLPs bearing EBOV GP^F535R^ are internalized and trafficked similarly to those harboring the wt GP, and while EBOV GP^F535R^ can also bind to NPC1 following cathepsin cleavage [34], it is unable to perform complete membrane fusion [9]. Therefore, these particles can be used to examine VLP accumulation at the site of fusion by microscopy. For these assays, we used HT1080 cells because their flat morphology allows for better visualization of VLPs in NPC1+ compartments. Importantly, EBOV entry into HT1080 cells is also blocked by the RTK inhibitors (S3 Fig). To determine if the inhibitors interfere with EBOV trafficking to NPC1+ compartments, HT1080 cells were incubated with EBOV GP^F535R^ VLPs in the presence of inhibitors or vehicle for 3 hours. Cells were fixed, immunostained for NPC1, and imaged by confocal microscopy. For these experiments, we used Akt Inhibitor VIII as a control because previous studies have shown an accumulation of EBOV in early endosomal compartments when Akt signaling was inhibited [17]. As we reported previously, we observed that a little over 10% of the VLPs colocalized with NPC1 in DMSO-treated cells (Fig 5B and 5C) [10]. However, when we blocked Akt signaling using Akt Inhibitor VIII, we found that colocalization of the VLPs with NPC1 was significantly reduced, indicating that they were not able to reach NPC1+ compartments (Fig 5C). Interestingly, when we compared the RTK inhibitors, we found that both SU11274 and NVP-ADW742 blocked VLP trafficking to NPC1 (Fig 5B and 5C). Surprisingly, however, Gefitinib treatment did not reduce VLP colocalization with NPC1, suggesting that the RTK inhibitors have different antiviral mode of actions. We also investigated colocalization of VLPs in vesicles that are NPC1+ and two-pore channel 2 (TPC2)+, which has been reported as the potential EBOV fusion-conducive compartment [9,35,36]; however, we did not observe a notable difference in VLP localization between vehicle- or Gefitinib-treated cells (S4A and S4B Fig). This further suggests that, unlike the other RTK inhibitors, Gefitinib does not block EBOV trafficking. Taken together, these data indicate that the RTK inhibitors have no effect on EBOV VLP internalization; instead, both SU11274 and NVP-ADW742 interfere with delivery of EBOV to its receptor.

### EGFR inhibitor treatment alters the biology and subvesicular localization of NPC1, rendering these compartments non-conducive to EBOV entry

**Fig 6:**
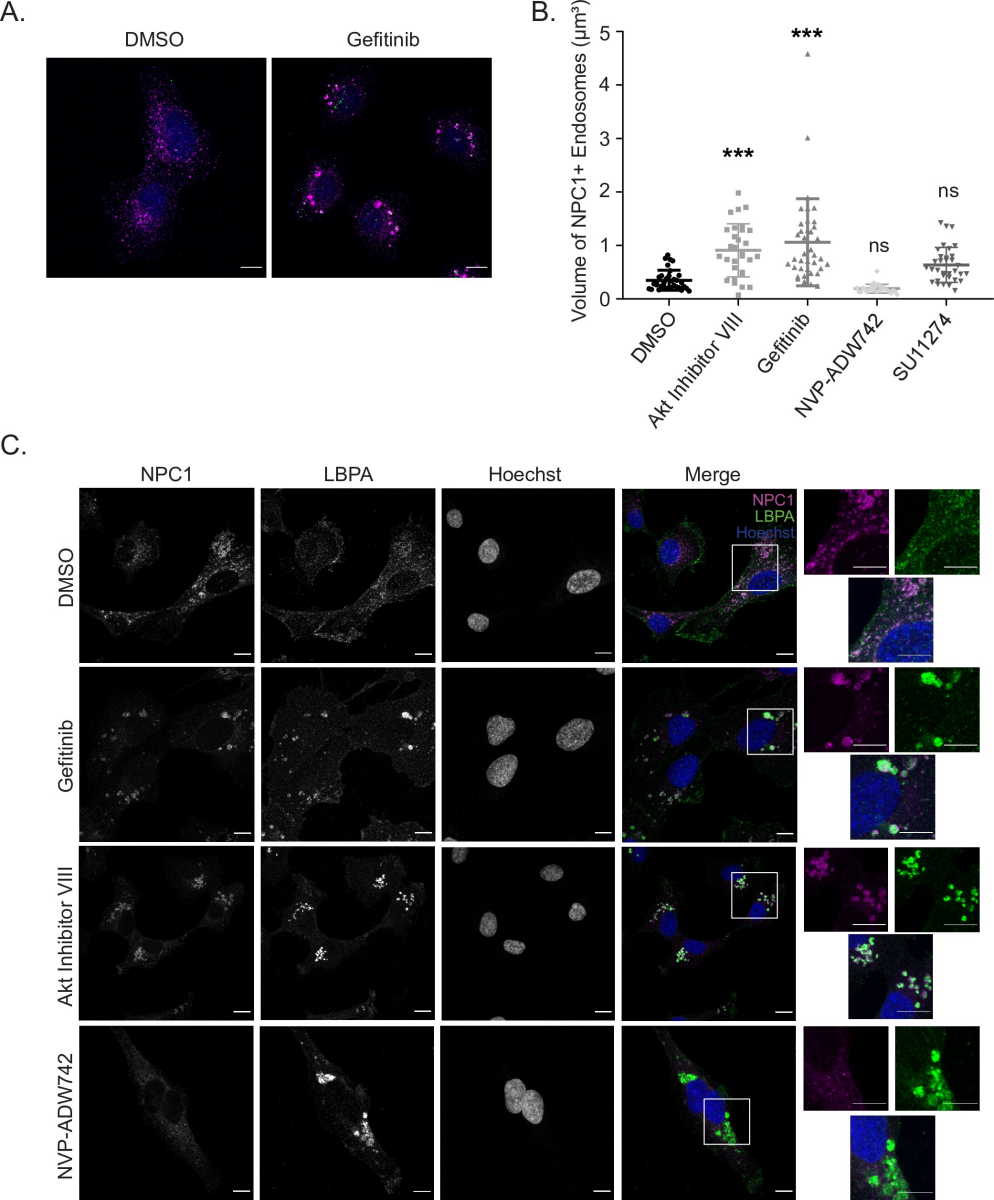
Gefitinib interferes with the biology of NPC1+ compartments. (A) Infection of HT1080 cells pre-treated with vehicle (DMSO, 0.1%) or Gefitinib (5 μM) with fluorescent VLPs (Green) harboring the fusion deficient EBOV GP^F535R^ for 3 h. 30 min prior to fixation, CMAC cytoplasmic dye (Blue) was added. Cells were then fixed, permeabilized, and stained for NPC1 (Magenta). Cells were imaged on an LSM800 confocal microscope (Zeiss). Images are displayed as maximum intensity z-projections, bar = 10 μm. (B) Average volume (μm^3^) of NPC1+ compartments per cell (of a minimum of 25 cells per condition) was determined by modeling these compartments using Imaris software (Bitplane). (C) HT1080 cells were treated with DMSO (0.1%), Gefitinib (5 μM), Akt Inhibitor VIII (10 μM), or NVP-ADW742 (2.5 μM) for 4 h. Cells were then fixed, permeabilized, and immunostained with rabbit anti-NPC1 and mouse anti-LBPA, followed by DY650-conjugated antiserum (Magenta) or AF555-conjugated antiserum (Green). Following immunostaining, cells were stained with Hoechst (Blue) and imaged on an LSM800 confocal microscope (Zeiss). Images are displayed as maximum intensity z-projections, bar = 10 μm. Data are representative of 3 independent experiments. * p < 0.05, ** p < 0.01, *** p < 0.001.

Because Gefitinib did not block EBOV trafficking to NPC1, we sought to analyze the NPC1+ compartment further. One striking phenotype observed in Gefitinib-treated cells was an enlargement of the NPC1+ compartments compared to those in vehicle-treated cells (Figs 5B, 6A and 6B). Quantitative measurement of the volume of these compartments indeed revealed that Gefitinib treatment, as well as treatment with Akt inhibitor VIII, led to a significant increase in their size (Fig 6B). However, neither NVP-ADW742 nor SU11274 affected the volume of the NPC1+ compartments. These results indicate that, although EBOV VLPs are delivered to NPC1+ compartments in Gefitinib-treated cells, these compartments are modified and are not conducive to entry. Interestingly, the blunt effect of blocking Akt directly, which caused both an enlargement of NPC1+ compartments and an EBOV VLP trafficking defect, resembles a combination of the phenotypes of Gefitinib, which also causes an enlargement of NPC1+ compartments, and those of SU11274/NVP-ADW742, causing trafficking defects (Figs 5C and 6B).

Previous studies have associated an enlargement of late endosomes/lysosomes with a decrease in cathepsin activity [37]. Because EBOV GP needs to be cleaved by cathepsin proteases to reveal its receptor binding domain [3,4,38,39], we sought to test whether the mechanism by which Gefitinib blocks EBOV entry involves a decrease in GP cleavage by cathepsins. To investigate this, we experimentally mimicked cathepsin cleavage of GP by pre-treating EBOV VLPs with thermolysin (S5A Fig) [40,41]. Entry of the uncleaved and cleaved VLPs was then measured in Vero cells treated with the RTK inhibitors or the cathepsin B inhibitor (Ca074-Me) as a control. As expected, Ca074-Me blocked entry of the uncleaved VLPs but was unable to inhibit entry of the pre-cleaved VLPs (S5B Fig). In contrast, we found that Gefitinib and the other RTK inhibitors blocked entry of both the uncleaved and cleaved VLPs (S5B Fig). Although these results do not completely rule out a defect in cathepsin activity, they suggest that another critical EBOV entry step or steps are inhibited in Gefitinib-treated cells.

Another factor that can lead to enlarged late endosomes/lysosomes is the accumulation of lipids such as cholesterol [42]. To investigate this, we used filipin to test whether cholesterol build-up is induced in Gefitinib-treated cells compared to the other RTK inhibitors and U18666A, a molecule that blocks EBOV entry and induces cholesterol accumulation in cells characteristic of Niemann-Pick C disease [4]. Interestingly, while increased filipin staining in Gefitinib-treated cells was noticeable, we also observed a similar phenotype in SU11274 and NVP-ADW742-treated cells (S6 Fig). This suggested that cholesterol build-up does not correlate with enlarged NPC1+ compartments nor the ability/inability of EBOV to traffic to NPC1. However, because there was no apparent trafficking defect in Gefitinib treated cells (Fig 5C), the accumulation of cholesterol in the presence of Gefitinib could be an indication that NPC1 function is altered. Therefore, we next sought to look more closely at the expression of NPC1 in intraluminal vesicle (ILV) containing endolysosomes, the typical site of cholesterol transport.

Normally, for low-density lipoprotein (LDL) associated cholesterol to be taken up by cells, it must be endocytosed and trafficked to NPC2+ and NPC1+ endolysosomes that are characterized by the presence of ILVs rich in the anionic lipid, lysobisphosphatitic acid (LBPA) [43,44]. To transport cholesterol from the endolysosome to the endoplasmic reticulum, NPC2 shuttles the cholesterol from the ILVs to NPC1 localized at the limiting membrane of the endolysosomes [43,44]. Using LBPA and NPC1 specific antibodies, we examined cells treated with vehicle, Gefitinib, or other EBOV inhibitors (Fig 6C). As expected, we observed that irrespective of the drug treatment, NPC1 can be found in both LBPA+ and − compartments. However, the enlarged NPC1+ vesicles found in Gefitinib- and Akt Inhibitor VIII-treated cells were almost all exclusively LBPA+ (Fig 6C). By examining the subvesicular localization of NPC1, we found that NPC1 staining surrounded the LBPA-containing vesicles in vehicle treated cells, suggesting the expected presence of NPC1 at the limiting membrane of these intracellular compartments (Fig 6C). Interestingly, the localization of NPC1 was substantially changed in the Gefitinib-treated cells; NPC1 seemed to be localized both at the limiting membrane as well as within the vesicles (Figs 6C and S7A). Further analysis revealed increased colocalization of NPC1 with LBPA (S7B Fig), suggesting that Gefitinib treatment causes a redistribution of NPC1 to intraluminal vesicles containing LBPA. Interestingly, this subvesicular localization of the EBOV receptor would presumably be incompatible with cytoplasmic delivery of the ribonucleocapsid. Taken together, our data suggest that treatment of cells with Gefitinib profoundly changes the biology of NPC1+ compartments, rendering them non-conducive to EBOV entry.

### EBOV VLPs induce Akt activation in a GP-independent manner

**Fig 7:**
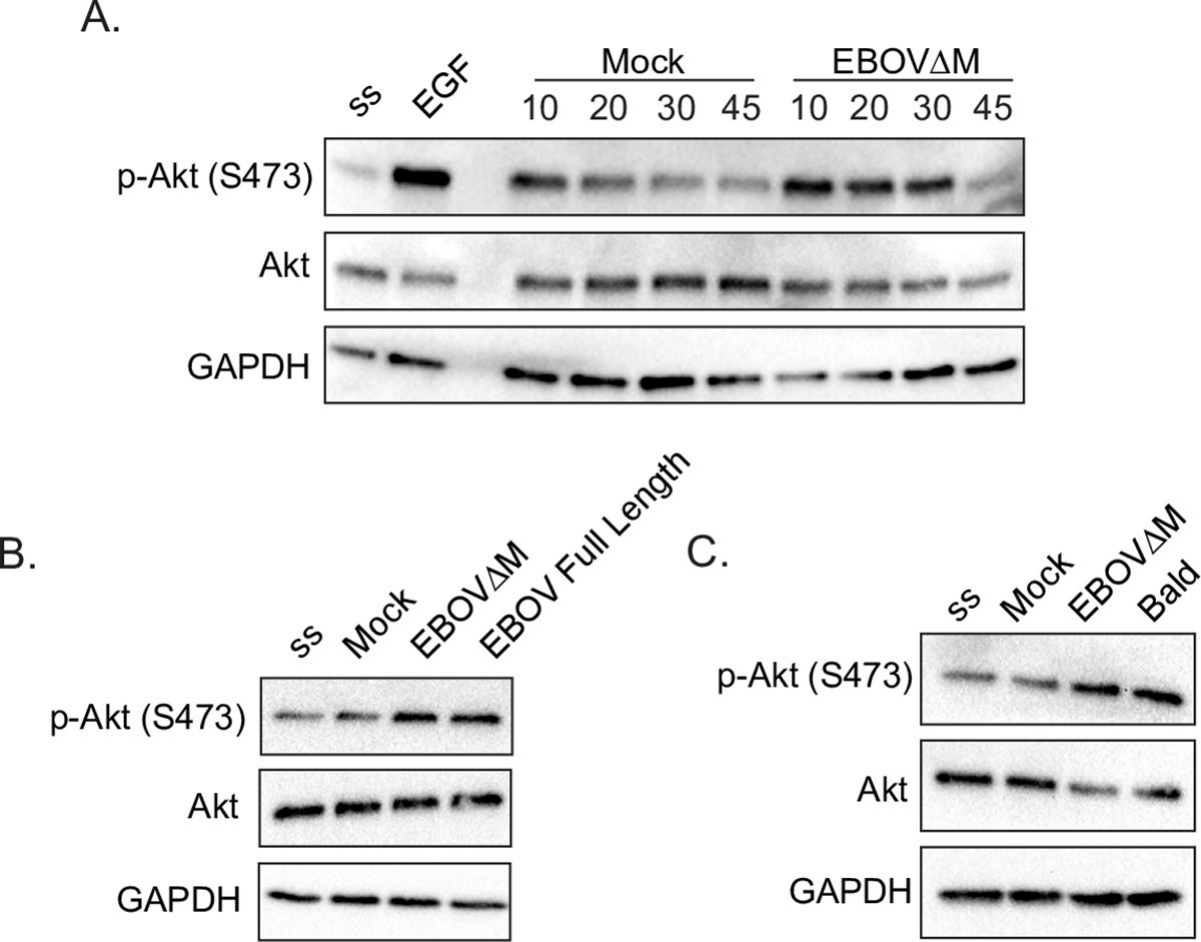
EBOV ΔM GP, EBOV Full Length GP, and Bald EboV VLPs all stimulate Akt phosphorylation in Vero cells. Vero cells were serum starved in HBSS for 1h, followed by stimulation with (A) EGF (50 ng/mL), Mock, or βlam EBOV ΔM GP VLPs for 10, 20, 30, or 45 min, (B) Mock, βlam EBOV ΔM GP, or βlam EBOV Full Length GP VLPs for 20 min., or (C) Mock, βlam EBOV ΔM GP VLPs, or Bald EBOV VLPs for 20min. Cells were lysed and phosphorylated Akt (p-Akt—S473), total Akt (Akt), and GAPDH were detected by immunoblot. Abbreviations are serum starved (ss), βlam EBOV ΔM GP VLPs (EBOV), βlam EBOV Full Length GP VLPs (Full Length). Data are representative of 3 independent experiments.

Our findings indicate that the RTK inhibitors have important consequences on EBOV trafficking or the biology of the NPC1+ compartments. A remaining question is whether EBOV activates RTK signaling or if viral entry relies on steady state cell signaling. As mentioned previously, studies using irradiated replicative EBOV have shown that EBOV triggers Akt phosphorylation, a pathway that is activated downstream of RTK stimulation [17]. To investigate a link between RTKs and Akt during EBOV entry, we first tested whether VLPs harboring EBOV GP can recapitulate the activation observed with irradiated EBOV. We performed a time-course experiment and probed for phosphorylated Akt after Vero cells were exposed to sucrose cushion purified EBOV VLPs or purified supernatant from mock-transfected producer cells (Fig 7A). We found that while the mock-transfected cell supernatants induced slight Akt activation at early time points, EBOV VLP-induced Akt activation was stronger and sustained for at least 30 minutes (Fig 7A).

Next, we sought to characterize further how VLPs activate Akt phosphorylation in cells. First, we compared Akt activation following incubation with VLPs harboring the full length or mucin deleted EBOV GP and found that both induced Akt phosphorylation to a similar extent in Vero cells (Fig 7B) and BMDMs (S8 Fig). In addition, the kinetics of Akt activation by full length EBOV GP VLPs followed closely that of its mucin-deleted counterpart (Figs 7A and S9). While these data indicate that the mucin region is not responsible for Akt activation, it is still unclear whether signaling is GP-dependent. Previous studies have shown that EBOV particles have exposed phosphatidylserine (PS) on the outer leaflet of the viral envelope, which has been shown to be actively induced by EBOV via PS flipping mechanisms that can be dependent on GP or VP40 [45,46]. To test whether induction of Akt phosphorylation can be GP-independent, we produced “bald” VLPs containing EBOV NP and GFP-VP40 but devoid of GP. These GFP+ VLPs can be readily detected by flow cytometry, and using fluorescently labeled annexin V, we confirmed exposure of PS at the surface of VLPs in the presence or absence of GP (S10 Fig). Importantly, all VLPs were able to activate Akt in cells (Fig 7C), strongly suggesting that EBOV can induce Akt phosphorylation in a GP-independent manner.

### The RTK inhibitors block EBOV-induced activation

**Fig 8:**
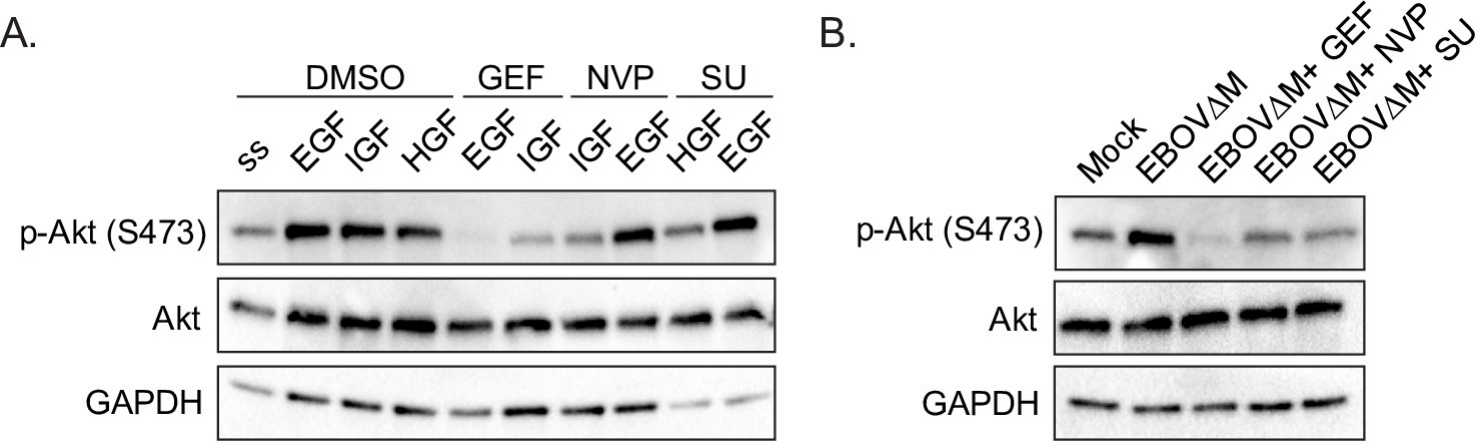
RTK inhibitors block EBOV VLP-induced Akt phosphorylation. Vero cells were pre-treated with Gefitinib (5 μM), SU11274 (2.5 μM), NVP-ADW742 (2.5 μM) or vehicle (DMSO, 0.1%) in serum-free HBSS for 1h. Cells were then stimulated with (A) EGF (50 ng/mL), IGF (50 ng/mL), or HGF (200 ng/mL) for 10, 20, 30, or 45 min, or (B) with Mock, βlam EBOV ΔM GP VLPs (MOI = 100) for 20 min. Cells were lysed and phosphorylated Akt (p-Akt—S473), total Akt (Akt), and GAPDH were detected by immunoblot. Abbreviations are serum starved (ss), βlam EBOV ΔM GP VLPs (EBOV), Gefitinib (GEF), NVP-ADW742 (NVP), and SU11274 (SU). Data are representative of 3 independent experiments.

Akt is known to be one of the signaling molecules downstream of RTK activation [47]. The resistance of EBOV GP-mediated entry to Gefitinib treatment in BMDMs, which do not express EGFR, provides evidence that the antiviral targets of these inhibitors are their respective RTKs (Fig 4A and 4B). To further confirm the specificity of the inhibitors at the concentrations used, we tested the effects of Gefitinib (EGFR inhibitor), NVP-ADW742 (IGF1R/InsR inhibitor), and SU11274 (c-Met inhibitor) on Akt activation induced by their respective growth factors. We found that at the EBOV IC90 concentrations, the c-Met and InsR/IGF1R inhibitors specifically blocked Akt phosphorylation when induced with the RTK growth factor ligand of their respective target (Fig 8A). Interestingly, while Gefitinib (the EGFR inhibitor), did abrogate EGF specific Akt phosphorylation, it reduced Akt phosphorylation to a lower level than the serum-starved control, suggesting that Gefitinib blocks both steady-state signaling in unstimulated cells as well as EGF-induced signaling (Fig 8A). In addition to EGF-, IGF-induced Akt phosphorylation was also reduced by Gefitinib (Fig 8A). Although this reduction could be due to EGFR transactivation by activated IGF1R [48], this potentially indicates that Gefitinib could have additional effects on downstream signaling following activation of other RTKs. Nonetheless, this experiment suggests that, at the concentrations used to block EBOV GP-mediated entry, the RTK inhibitors block Akt signaling in a target specific manner.

Given that RTK inhibitors block EBOV entry and that EBOV VLPs induce Akt phosphorylation, a straightforward hypothesis is that RTK activation is responsible for Akt signaling induced by EBOV. To test this, we exposed Vero cells to EBOV VLPs in the presence or absence of the RTK inhibitors and found that all inhibitors were able to block EBOV VLP-induced Akt phosphorylation (Fig 8B). These results suggest that EBOV VLPs can indeed activate Akt phosphorylation during entry via RTK-dependent signaling cascades. Taking our conclusions in conjunction with the findings of Saeed et al. [17] examining the role of Akt in EBOV trafficking, these results serve to further suggest that EBOV VLPs can induce RTK-dependent activation of Akt to promote trafficking to entry-conducive NPC1+ compartments.

## Discussion

EBOV entry into host cells is a multistep process that requires internalization by macropinocytosis and trafficking through the endosomal pathway to reach vesicular compartments containing triggering factors such as cathepsin proteases and NPC1 [2–4,7,8,10,38,49]. While the specific host factors involved in regulating EBOV trafficking, such as the HOPS and PAS complexes, are becoming increasingly well-characterized, it is still unclear whether EBOV particles are passive passengers of the endolysosomal system or hijackers–actively directing their delivery to entry-conducive compartments. In this study, we show that EBOV uses and activates signaling pathways to promote its delivery to NPC1+ compartments that can facilitate delivery of the ribonucleocapsid to the cytoplasm.

By screening a library of kinase inhibitors, we identified specific and common signaling pathways required for EBOV and/or MARV entry (Fig 1C). Previous studies have also uncovered the importance of cellular kinases in the different steps of the EBOV entry. For example, studies have suggested that AMPK [22] and diacylglycerol (DAG) kinases [32] play a role in the macropinocytic uptake of EBOV. Interestingly, the AMPK inhibitor, Dorsomorphin, was also identified in our screen; although it mostly affected MARV GP-mediated entry, it also slightly reduced transduction by EBOV pseudotypes (Fig 1C). Whether AMPK or DAG kinases are activated during entry or if the virus relies on steady state signaling through these kinases for macropinocytic uptake remains to be determined. However, because small-molecule inhibitors of both AMPK and DAG kinases are also capable of blocking high molecular weight dextran, it is likely that treatment with these inhibitors results in a general block of the macropinocytic uptake process, whether EBOV is present or not. In addition to AMPK and DAG kinases, a previous study utilized G-protein-coupled receptor (GPCR) antagonists to reveal that GPCR signaling might be involved in a post-attachment entry step [50]. However, it is unclear if the antiviral target of these GPCR inhibitors is a host factor or if the inhibitors act via direct binding to EBOV GP [51]. Interestingly, in our study, EBOV GP-mediated entry was blocked by FTY720 (Fig 1C), an antagonist of the sphingosine-1-phosphate (S1P) receptor GPCR [52]. S1P, the GPCR ligand, is generated through phosphorylation of sphingosine by sphingosine kinases; interestingly, we also identified a sphingosine kinase inhibitor (PF-543) in our screen (Fig 1C) [53]. The identification of both FTY720 and PF-543 raises the interesting hypothesis that the SK-S1PR axis may also play a role in EBOV GP-mediated entry. More work needs to be done to test this hypothesis and to elucidate whether these signaling pathways are activated by the virus to promote entry.

In our small-molecule screen, we also identified RTKs as a common and overrepresented signaling pathway among our hits (Fig 1D). Inhibitors of EGFR, c-Met, or InsR/IGF1R blocked EBOV GP-mediated entry in multiple cell lines in addition to replicative EBOV growth. While previous studies have suggested that EGFR and Ephrin receptor signaling is important for Ebola virus infection, our study was the first to provide a detailed mechanism of action [54–56]. Since RTKs have been reported to facilitate the macropinocytosis of other viruses, we sought to determine if this is true for Ebola virus as well [57]. Interestingly, we found that the RTK inhibitors did not interfere with internalization, but rather, the c-Met and InsR/IGFR inhibitors prevented the delivery of viral particles to NPC1+ compartments (Fig 5B and 5C). Each of these inhibitors also blocked EBOV-induced Akt signaling during entry (Fig 8B). Since Akt signaling has also been characterized to be important for EBOV trafficking [17], our data suggests that Akt signaling may be initiated through RTK dependent signaling pathways. Additionally, production of PtdIns(3,5)P2 via PIKfyve is also enhanced by EBOV, and previous work has shown that EGF stimulation leads to an increase in PIKfyve activity via Akt-mediated phosphorylation [16]. Interestingly, chemical inhibition of Akt or PIKfyve leads to a block in EBOV VLP trafficking to NPC1+ compartments [10,11,17]. While a link between Akt and PIKfyve is implied by the literature, the mechanisms by which these pathways are activated by EBOV remain unknown. It is tempting to speculate that one of the roles of the EBOV induced RTK-Akt signaling is to activate PIKfyve for efficient delivery to NPC1+ endolysosomes. More work needs to be done to test this hypothesis.

Similar to the c-Met and IGF1R/InsR inhibitors, the EGFR inhibitor also led to a block in EBOV-induced Akt activation (Fig 8B). Surprisingly, however, treatment with this inhibitor did not impair the delivery of EBOV VLPs to NPC1+ compartments (Fig 5B and 5C). Since EBOV was able to reach the NPC1+ compartments following inhibitor treatment but was unable to successfully enter cells, this implied that Gefitinib treatment renders the NPC1+ compartments non-conducive to entry. Interestingly, we observed that Gefitinib-treated cells had enlarged NPC1+ endosomes (Fig 6A and 6B) and exhibited a distinctive NPC1 expression pattern, with NPC1 appearing inside the endosomes in addition to its expected localization at the limiting membrane (Fig 6C). The redistribution of NPC1 also explains the apparent accumulation of cholesterol in these vesicles (S6 Fig), since it must be shuttled from LBPA rich intraluminal vesicles by NPC2 to NPC1 localized at the limiting membrane of the endolysosomes for transport to the endoplasmic reticulum [43,44]. Because EBOV VLPs were observed to colocalize with NPC1 inside the enlarged endosomes of Gefitinib-treated cells (Fig 5B and 5C), we speculate that, in Gefitinib-treated cells, fusion may occur in these NPC1+ intraluminal vesicles instead of at the limiting membrane of the endosome, thus preventing the ribonucleocapsid from reaching the cytoplasm. Interestingly, our studies also revealed that, in addition to blocking EGF- and EBOV-induced Akt activation, Gefitinib reduced Akt phosphorylation below that observed in serum-starved cells (Fig 8A and 8B). This finding suggests that the biology of the NPC1 compartment is modulated by steady state signaling and may not be dependent on EBOV entry. Future studies will aim to further characterize the NPC1+ compartments after Gefitinib treatment.

To begin dissecting how signaling pathways are activated during filoviral entry, here we provide evidence that EBOV-induced Akt activation is RTK dependent and can occur in the absence of GP (Figs 7C and 8B). With regards to filovirus induced signaling pathways, previous studies have shown that cells, especially immune cells, can become activated after exposure to EBOV [58–60]. For instance, in dendritic cells, EBOV VLPs activate NF-κB and MAPK signaling in a EBOV GP-dependent manner, and that this requires the mucin domain for full activation [59]. Additionally, other studies have shown that the presence of EBOV GP on VLPs was required to engage TLR4 in multiple cell lines [58]. Further, EBOV can also activate lymphocytes via binding to TIM-1, presumably via virus exposed PS [60,61]. While these studies clearly demonstrate that signaling pathways can be activated in response to EBOV, we chose to focus on identifying pathways that are activated by EBOV to promote entry. We demonstrated that EBOV can activate Akt both in the presence or absence of GP on VLPs (Fig 7), suggesting that a lipid on the envelope may be required. It is well-established in the literature that PS on the surface of enveloped viruses, including EBOV, can bind to cell surface PS receptors and mediate attachment and internalization of virions [62,63]. The receptor tyrosine kinases Tyro3, Axl, and Mer (TAM family) are three examples of such PS receptors; for these, binding of PS is mediated by Protein S or Gas6 [61,64]. RTKs, including those of the TAM family, can undergo unconventional heterodimerization among their different classes. More specifically, it has been shown in multiple cell lines that Axl and EGFR can heterodimerize and that interaction with Axl can drive EGFR signaling through the PI3K/Akt pathway, even in the presence of an EGFR blocking antibody [65,66]. Interestingly, for other enveloped viruses that utilize the TAM family of RTKs during entry, evidence suggests that the protein tyrosine kinase domain of Axl is dispensable for enhancement of virus attachment and internalization; however, it has also been shown that subsequent infection is impaired when there is no kinase activity [67,68]. In conjunction with our data, it is tempting to speculate that heterodimerization of RTKs of different classes, driven by PS on the viral envelope and the TAM RTKs, may activate signaling cascades through the PI3K/Akt pathway and promote viral entry. Notably, a model requiring hetero-oligomerization of multiple RTKs would explain the dominant negative effect of each RTK inhibitor on viral entry. More work needs to be done to test this hypothesis and to determine if c-Met, IGF1R, and InsR can also heterodimerize with the TAM family of RTKs.

While this manuscript was in peer-review, Kuroda et al. reported a role for HER2 and other RTKs in EBOV infection [69]. Interestingly, they also came to a similar model for Akt activation via TAM RTKs. Their study mainly focused on the role of HER2 in entry and proposed that it is involved in macropinocytic uptake. This proposed mechanism of action was based off the findings that overexpression of HER2 leads to increased dextran uptake and that viral particles colocalized with HER2/TYRO3 complexes at the cell surface but not once virions were internalized. In our study, inhibition of RTK signaling had no effect on EBOV internalization. This discrepancy could be explained by the use of HT1080 cells for many of our experiments, which are known to be HER2 negative or express a very low level of this RTK [70,71]. Collectively, the two studies highlight differential usage of RTK signaling for entry depending on the expression profiles in the target cells. Future work will aim at dissecting the contribution of each RTK on the different steps of the filovirus entry pathway.

In conclusion, the characterization of host trafficking factors and signaling pathways activated during viral entry are important to further our understanding of EBOV infection and provide potential targets for antiviral therapies. In our study, we identified RTK inhibitors, some of which are FDA-approved drugs, that could be used to block entry by all known pathogenic filoviruses. Since all filoviruses known to date, even Měnglà virus recently discovered in bats in China [72], use NPC1 as their entry receptor, these inhibitors have the potential to be effective antiviral agents against all filoviruses and development of such therapies may allow us to be prepared for future outbreaks sparked by emerging filoviruses.

## Materials and methods

### Ethics statement

All animal procedures were approved by the University of Ottawa Animal Care Committee (protocol number BMI-1863), which are in compliance with the standards of the Canadian Council on Animal Care (CCAC), and Ontario’s Animals for Research Act and its regulations.

### Cell lines and cell culture

Vero cells (ATCC) and HT1080 cells (ATCC) were cultured in Minimum Essential Medium (MEM, Sigma,) and Human Embryonic Kidney HEK293T (ATCC) cells were cultured in Dulbecco’s Modified Eagle Medium (DMEM, Wisent). All culture media were supplemented with 10% Fetal Bovine Serum (FBS, Sigma), 0.3 mg/mL L-glutamine, 100 U/mL penicillin, and 100μg/mL streptomycin (Wisent). Cells were maintained at 37°C in 5% CO_2_ at 100% relative humidity.

BMDMs were isolated from C57Bl/6J mice (stock no. 00064, originally purchased from Jackson Laboratories and maintained as a colony at the University of Ottawa) and differentiated as previously described [73]. Briefly, bone marrow cells were seeded in DMEM supplemented with 10% FBS (Wisent), penicillin, and streptomycin (Hyclone, GE healthcare). L929-conditioned media (20%) was utilized for macrophage differentiation, and Roswell Park Memorial Institute (RPMI, Wisent) media supplemented with 10% FBS (Sigma-Aldrich), 0.3mg/mL L-glutamine, 100 U/mL penicillin, and 100 μg/mL streptomycin (Wisent) was utilized for seeding and subsequent experiments.

### Small molecule inhibitors, antibodies and plasmids

SU11274, NVP-ADW742, Akt Inhibitor VIII, and 5-(N-ethyl-N-isopropyl)-Amiloride (EIPA) were all purchased from Cayman Chemical and Gefitinib was purchased from ApexBio. Stock solutions were prepared in DMSO, aliquoted, and stored at -80°C until use. Filipin III from *Streptomyces filipinensis* (F 4767) was purchased from Sigma and resuspended in DMSO prior to use.

Anti-Akt (9272S), anti-phospho-Akt S473 (92721S), and anti-mouse IgG HRP-linked (7076S) antibodies were purchased from Cell Signaling Technology. GAPDH (ab8245), EGFR (ab52894), Met (ab51067), NPC1 (ab134113), InsR (ab69508), and DY650 sheep anti-rabbit (ab96926) antibodies were purchased from Abcam. The LBPA (Z-PLBPA) antibody was purchased from Echelon Biosciences. The goat anti-Rabbit IgG HRP-linked (31460) and donkey anti-mouse Alexa Fluor 555 antibodies (A-31570) were purchased from ThermoFisher. The pan-filovirus anti-GP antibody (21D10) was purchased from IBT Bioservices.

Plasmids encoding the different virus glycoproteins (EBOV Δmucin GP, BDBV Δmucin GP, SUDV Δmucin GP, MARV GP, EBOV full-length GP, EBOV Δmucin GP^F535R^, and VSV G), pCAGGS, MLV gag/pol packaging plasmid, and the MLV retroviral vector encoding LacZ were kind gifts of Dr. James Cunningham, Brigham and Women’s Hospital. Plasmids encoding the EBOV NP and EBOV VP40-β-lactamase were kind gifts of Dr. Lijun Rong, University of Illinois. The EBOV VP40-mCherry plasmid was a gift from Dr. Judith White, University of Virginia (Addgene plasmid #74421) [74]. The GFP-TPC2 plasmid was a gift from Dr. Santiago Di Pietro, Colorado State University (Addgene plasmid #80153).

### Murine leukemia virus pseudotypes and viral-like particles production

Murine leukemia virus (MLV) pseudotypes were prepared by co-transfecting 293T cells with the MLV packaging plasmid gag-pol, a MLV retroviral vector encoding LacZ, and a plasmid encoding the glycoprotein of interest (EBOV Δmucin GP, MARV GP, or VSV G) at a 1:1:1.25 ratio respectively. Similarly, EBOV viral-like particles (VLPs) were prepared by co-transfecting 293T cells with plasmids encoding the EBOV nucleoprotein (NP), EBOV VP40 fused to β-lactamase (βlam), mCherry or GFP, and pCAGGS (Bald VLPs) or the viral glycoprotein of interest (EBOV Δmucin GP, EBOV Δmucin GP^F535R^, EBOV Full Length GP, SUDV Δmucin GP, BDBV Δmucin GP, MARV GP, or VSV G) at a 1:1:1.25 ratio. For the mock control, the pCAGGS plasmid was transfected instead. Transfections were performed using the jetPRIME transfection reagent (Polyplus Transfection) according to the manufacturers protocol. For MLV pseudotypes, VLPs, and mock, supernatants were harvested 48, 72, and 96 h post-transfection followed by concentration by ultracentrifugation (20,000 RPM, 4°C, 1.5h, Beckman Coulter Optima XPN-100, SW32Ti rotor) through a 20% (*w/v*) sucrose cushion. Pellets were re-suspended in PBS, aliquoted, and stored at -80°C.

### MLV transduction and VLP entry assays

For MLV pseudotype transduction assays, Vero cells were seeded and grown to 60% confluency in white 96 well plates. 30 minutes prior to infection with MLV pseudotypes, the cells were pre-treated with inhibitors or vehicle (DMSO) in serum-free MEM containing 5 μg/mL polybrene. Four hours post-infection, the media was replaced with phenol-red free DMEM containing 15 mM NH_4_Cl and supplemented with 10% FBS (Sigma), 0.3 mg/mL L-glutamine, 100 U/mL penicillin, and 100μg/mL streptomycin (Wisent). Twelve hours later, the media was again replaced with phenol red free DMEM as described above, but without NH_4_Cl, and cells were incubated an additional 48 hours. LacZ+ cells were quantified using the Beta-Glo Assay System (Promega) following the manufacturers protocol. Luminescence was measured using a Synergy Neo2 Multi-Mode plate reader (BioTek).

For VLP infection assays, Vero, HT1080 or BMDMs were seeded and grown to 90% confluency in 48 well plates. Prior to virus addition, the cells were pre-treated with inhibitor or vehicle in serum-free MEM (for Vero and HT1080 cells) or serum-free RPMI (for BMDMs). 30 minutes later, βlam VLPs were added at a MOI between 0.2 and 0.4. Three hours post-infection, cells were loaded with a β-lactamase cleavable FRET substrate, CCF2-AM (ThermoFisher), according to manufacturer’s protocol and supplemented with 15 mM NH_4_Cl. One to two hours later, cells were trypsinized and prepared for analysis by flow cytometry (FACSCelesta, BD Biosciences). Analysis was performed using FlowJo software (BD Biosciences) and infection was quantified by using uninfected controls to assess the percentage of cells that underwent a shift from 530 nm to 460 nm emission, representing cleaved CCF2.

### Small molecule inhibitor screen

The small molecule inhibitor screen was performed using MLV pseudotypes (as described above) on a 418 compound L1200 kinase inhibitor library (Selleckchem). Metabolic activity was assessed in parallel at the equivalent time point of 12 hours post infection (cells were not infected for metabolic activity measurement) using the CellTiter-Glo assay system (Promega) according to manufacturer’s protocol. Luminescence was measured for infection and metabolic activity assays using a Synergy Neo2 Multi-Mode plate reader (BioTek). Relative percent infection or relative percent viability was then calculated based on luminescence from wells that contained vehicle alone.

The mean of each technical duplicate was calculated and, from these, the mean of each biological triplicate was calculated. Using the mean metabolic activity normalized to that of the vehicle treated cells, we set a cutoff of 80% and eliminated the data from these compounds (9 compounds). Data from the compounds with negative mean values were also eliminated (10 compounds). The ratio between the mean of each virus versus the mean of VSV was then determined. Using the triplicates, p-values (t-test) between EBOV/MARV and VSV were calculated. Using the ratio and the p-value, volcano plots were generated. Cutoffs of 2-fold and 0.05 for the ratios and p-values, respectively, were used. Heatmaps were also generated using log2 ratios.

### Replication-competent virus growth assay

Vero cells were seeded in clear bottom, black well tissue culture plates (Corning). Cells at 80% confluence were treated with different concentrations of the RTK inhibitors and infected with EBOV (strain Mayinga), expressing enhanced-GFP, at a multiplicity of infection of 0.1. Virus growth was assessed by measurement of GFP at different time-points using a BioTek Synergy/HTX plate reader with excitation at 485nm and emission at 516nm. Experiments with replication-competent EBOV were performed in the Biosafety Level 4 facility at the National Microbiology Laboratory at the Public Health Agency of Canada in Winnipeg.

### Pre-cleaved virus assay

βlam VLPs harboring EBOV Δmucin GP were incubated in 0.2 mg/mL thermolysin (Sigma) or PBS for 10, 20, and 30 minutes at 37°C to determine the optimal length of thermolysin incubation. Thermolysin activity was quenched by incubation in phosphoramidon (Sigma, 500 μM) on ice for 10 minutes. A portion of the samples were aliquoted and stored at -80°C for future use, while another portion was used to prepare lysates for immunoblotting. The resulting PVDF membrane was probed with a pan-filovirus anti-GP antibody. The 20 minute time point was selected to be optimal and VLP entry experiments were performed with the pre-cleaved virus as described above.

### Internalization assay

Vero cells were seeded and grown to 90% confluency in 48 well plates. Cells were pre-treated with serum-free MEM (Vero) or RPMI (BMDM) containing inhibitor or vehicle (DMSO) for 30 minutes at 37°C followed by 15 minutes at 4°C. The mCherry VLPs were added on ice and attached to the surface of the cells by centrifugation at 300 x g for 30 minutes at 4°C. Following centrifugation, cells were washed 3 times with ice-cold PBS, pre-warmed serum-free media containing inhibitor or vehicle was added, and cells were then incubated at 37°C for 1 h. In parallel, one set of vehicle alone samples was not moved to 37°C but rather kept at 4°C following the cold PBS washes to serve as a no internalization control. After 1 h, the cells were washed with cold PBS and incubated at 4°C with 0.5% Trypsin-EDTA (Gibco*)* for 30 min. Samples were prepared and analyzed by flow cytometry (Celesta, BD Biosciences). Analysis was performed using FlowJo (BD Biosciences) and mCherry mean fluorescence intensity was determined for each sample.

### NPC1 and NPC1/TPC2 VLP colocalization assays

HT1080 cells were seeded onto coverslips and grown to 50% confluency. Cells were pre-treated in serum-free MEM with inhibitor or vehicle for 1 h followed by addition of mCherry VLPs harboring the fusion deficient EBOV Δmucin GP^F535R^ and incubated at 37°C for 3 h. 30 minutes before fixation, CellTracker Blue CMAC dye (ThermoFisher) was added according to manufacturer’s protocol. 3h post infection, cells were fixed with formalin, permeabilized with 0.5% triton X-100, and blocked with 20% FBS in PBS for 30 minutes. Cells were incubated with a NPC1 primary antibody (1:70) followed by a DY650 secondary antibody (1:400), and mounted with PermaFluor Aqueous Mounting medium (ThermoFisher). Cells were imaged by confocal microscopy (LSM800 AxioObserver Z1, Zeiss) using a 63x / 1.4NA oil Plan Apochromat objective. Fifteen z-stacks were acquired per image, with a pixel size of 0.1 μm.

Image analysis was performed using Imaris software v. 8.4.2 (Bitplane). In brief, VLPs were modelled as spots and each cell was modeled as a surface based on the CMAC cytoplasmic stain. Then, the number of spots per cell was determined and the modeled spots were assigned colocalization values based on intensity correlation to NPC1. Colocalization thresholds were set manually by determining the minimum mean fluorescence intensity value in the NPC1 channel that corresponded to a colocalized VLP, modeled as a spot. Intensity thresholds were set for each experiment but kept constant between experimental conditions. The percentage of VLPs colocalizing with NPC1 was then determined by dividing the number of spots above the colocalization threshold by the total number of spots per cell.

To analyze NPC1+ compartment size, the far-red channel (NPC1) was modelled as a surface with thresholds manually set per image. To avoid bias, image files were re-named, and the surface modeling was performed blind. The surfaces were then divided into cells based on the cells that were modeled previously for the NPC1 colocalization analysis. The statistics for surface volume was exported and the average volume of NPC1+ compartments per cell was determined.

For the NPC1/TPC2 colocalization assay, HT1080 cells were transfected with a GFP-TPC2-encoding plasmid prior to re-seeding and infection with EBOV VLPs as described above. NPC1 immunofluorescence was performed as described above with the exception that cells were stained with Hoechst (Invitrogen) instead of CMAC. Similar to the NPC1 colocalization assays, image analysis was performed using Imaris software v. 8.4.2 (Bitplane). VLPs were modeled as spots and each cell was modeled as a surface based on background immunofluorescence seen by bumping up the intensity threshold for the NPC1 channel. Intensity thresholds were set for NPC1 and TPC2 (with a certain intensity rating representing the presence of either marker), and the number of VLPs in [NPC1+, TPC2+], [NPC1-, TPC2+], or [NPC1+, TPC2-] were determined and expressed as percentages compared to the total number of VLPs in each cell.

### LBPA assay

HT1080 cells were seeded onto coverslips, grown to 50% confluency, and incubated at 37°C for 4h with inhibitor or vehicle in serum free MEM. Cells were fixed with formalin and blocked with 20% FBS in 0.05% saponin-PBS (Saponin, EMD Millipore). Cells were incubated in 0.05% saponin-PBS with a LBPA primary antibody (1:100) and NPC1 primary antibody (1:70), followed by an AF555 (1:400) and DY650 secondary antibody (1:400). Cells were stained with Hoechst and then mounted with PermaFluor Aqueous mounting medium (ThermoFisher). Imaging was performed by confocal microscopy (LSM800 AxioObserver Z1, Zeiss) using a 63x / 1.4NA oil Plan Apochromat objective. An average of twenty z-stacks were acquired per image, with a pixel size of 0.1 μm. Image analysis was performed using Imaris software v. 9.6.0 (Bitplane). In brief, cells were modeled as surfaces and masks were created to separate each cell into a channel. Intensity based thresholds for the NPC1 and LBPA channels were determined manually (and kept consistent for each experiment across conditions) and Pearsons’s coefficient was calculated for each cell using the colocalization module.

### Filipin III staining

HT1080 cells were seeded onto coverslips, grown to 50% confluency, and incubated at 37°C for 4h with inhibitor or vehicle in serum free MEM. Cells were fixed with formalin, incubated with Filipin III (50 μg/mL in PBS) for 2h at RT, and coverslips mounted with PermaFluor Aqueous mounting medium. Cells were imaged by confocal microscopy (LSM800 AxioObserver Z1, Zeiss) using a 63x / 1.4NA oil Plan Apochromat objective. An average of twenty z-stacks were acquired per image, with a pixel size of 0.1 μm.

### Immunoblots

Vero cells in growth phase were seeded and grown to 50% confluency in 12 well plates. Cells were washed 3 times with pre-warmed Hanks’ Balanced Salt Solution (HBSS, Sigma) and pre-treated for 1h in serum-free HBSS containing the appropriate RTK inhibitor or vehicle (DMSO). After one hour, cells were stimulated in serum-free HBSS for either the indicated time point or 20 min with EGF (50 ng/mL), IGF (50 ng/mL), HGF (200 ng/mL), βlam VLPs harboring EBOV GP at a MOI of 100, or mock at the same volume as the volume of VLPs. Cells were then washed once with cold PBS and lysed in cold lysis buffer (1% Triton X-100, 0.1% IGEPAL CA-630, 150mM NaCl, 50mM Tris-HCl, pH 7.5) containing protease and phosphatase inhibitors (Cell Signaling). Proteins in cell lysates were resolved on SDS-polyacrylamide gels (Bio-Rad) and transferred to polyvinylidenedifluoride (PVDF) membranes. Membranes were blocked for 1h at RT with blocking buffer (5% skim milk powder dissolved in 25mM Tris, pH 7.5, 150mM NaCl, and 0.1% Tween-20 [TBST]) containing sodium orthovanadate (Na_3_VO_4_, 1mM, Alfa Aesar) and sodium fluoride (NaF, 10mM, VWR). PVDF membranes were then incubated overnight at 4°C with the appropriate primary antibody in 5% bovine serum albumin (BSA, Sigma) in TBST containing Na_3_VO_4_ and NaF. Blots were then washed in TBST and incubated with HRP-conjugated secondary antibody for 1h at room temperature. PVDF membranes were washed again, incubated in chemiluminescence substrate and imaged using the ChemiDoc XRS+ imaging system (Bio-Rad). In some instances, the same membrane was stripped and re-probed for total Akt.

### Annexin V staining and flow cytometry analysis of VLPs

EBOV VLPs were produced as described above. VLP concentration was determined using a Zetaview nanoparticle tracking instrument (ParticleMatrix). Then, the VLPs were diluted to a concentration of 10^9^ particles/mL in Annexin V Binding Buffer (ABB: 10mM Hepes pH 7.4, 140mM NaCl, 2.5mM CaCl_2_, filtered with a 0.1μm pore). To stain VLPs, 5uL of Annexin V-PE (BD Biosciences) was added to 100uL of VLP in ABB and incubated in the dark for 30 minutes at 4°C. The stained sample was then diluted in ABB at a 1:500 ratio in preparation for sample acquisition using a CytoFLEX S (Beckman Coulter). The FITC channel (525/40) and PE channel (585/42) were used to detect VLPs and AnnexinV, respectively. Samples were collected at a flow rate of 10uL/min with cytometer calibration and settings previously described [75]. Data analysis was performed using FlowJo (Version 10.7.0).

## Supporting information

S1 Data Raw data from small molecule kinase screen. (XLSX)

S1 Table Hits from small molecule kinase screen for EBOV. (DOCX)

S2 Table Hits from small molecule kinase screen for MARV. (DOCX)

S1 Fig Effect of a panel of RTK inhibitors on EBOV GP-mediated entry. Vero cells were exposed to βlam VLPs harboring the EBOV GP or VSV G in the presence of vehicle (DMSO, 0.1%) or the indicated RTK inhibitor at 1 μM. Viral entry was detected via flow cytometry after loading cells with the βlam substrate, CCF2, and quantifying the percentage of cells with cleaved CCF2. Data are expressed as percentages of inhibitor treated cells relative to vehicle alone. Data are representative of 3 independent experiments. * p < 0.05, ** p < 0.01, *** p < 0.001.
(TIF)

S2 Fig RTK inhibitors block entry of EBOV ΔM GP and EBOV Full Length GP to the same extent. Vero cells were exposed to βlam VLPs harboring VSV-G, EBOV ΔM GP or the EBOV Full Length GP in the presence of vehicle (DMSO, 0.1%), Gefitinib (1 μM), SU11274 (500 nM), or NVP-ADW742 (500nM). Entry was detected via flow cytometry after loading cells with βlam substrate (CCF2) and measuring the percentage of cells with cleaved CCF2. Data are expressed as percentages of inhibitor treated cells relative to vehicle alone. Data are representative of 3 independent experiments. Students t-test was performed to compare % entry for EBOV ΔM GP and EBOV Full Length GP.
(TIF)

S3 Fig RTK inhibitors block filovirus entry in HT1080 cells. HT1080 were exposed to βlam VLPs harboring the EBOV GP or VSV G in the presence of vehicle (DMSO, 0.1%) or increasing concentrations of Gefitinib, SU11274, or NVP-ADW742. Entry was detected via flow cytometry after loading cells with βlam substrate (CCF2) and measuring the percentage of cells with cleaved CCF2. Data are expressed as percentages of inhibitor treated cells relative to vehicle alone. Data are representative of 3 independent experiments.
(TIF)

S4 Fig Localization of EBOV VLPs in NPC1+ TPC2- compartments does not explain the antiviral activity of Gefitinib. (A-B) HT1080 cells that were transfected with GFP-TPC2 (Red) and pre-treated with vehicle (DMSO, 0.1%) or Gefitinib (5 μM) were exposed to fluorescent VLPs (Green) harboring the fusion deficient ΔM GP^F535R^ for 3 h. Cells were then fixed, permeabilized, immunostained with rabbit anti-NPC1 and DY650-conjugated antiserum (Magenta), and Hoechst (Blue). Cells were imaged on an LSM800 confocal microscope (Zeiss). Images in (A) are displayed as maximum intensity z-projections, bar = 10 μm. (B) Colocalization between VLPs and NPC1 and/or TPC2 were analyzed using Imaris software (Bitplane). Data are representative of 3 independent experiments. * p < 0.05, ** p < 0.01, *** p < 0.001.
(TIF)

S5 Fig RTK inhibitors are sensitive to entry by pre-cleaved EBOV VLPs. (A) βlam VLPs harboring the EBOV ΔM GP were incubated either with thermolysin (0.2 mg/mL) (Pre-cleaved) or PBS (Mock) for 10, 20, or 30 minutes prior to addition of phosphoramidon (500 μM). Lysates were prepared and immunoblotted for EBOV GP. (B) Pre-cleaved or mock virus that was incubated with thermolysin or PBS for 20 minutes was used to infect Vero cells treated with vehicle (DMSO, 0.1%), Ca074-Me (20 μM), Gefitinib (5 μM), SU11274 (2.5 μM), or NVP-ADW742 (2.5 μM). Entry was detected via flow cytometry after loading cells with βlam substrate (CCF2) and measuring the percentage of cells with cleaved CCF2. Data are expressed as percentages of inhibitor treated cells relative to vehicle alone. Data are representative of 3 independent experiments.
(TIF)

S6 Fig Treatment of cells with RTK inhibitors leads to cholesterol accumulation in cells. HT1080 cells were treated with vehicle (DMSO, 0.1%), Gefitinib (5 μM), SU11274 (2.5 μM), NVP-ADW742 (2.5 μM), Akt Inhibitor VIII (10 μM), or U18666A (5 μM) for 4 h. Cells were then fixed, stained with Filipin III, and imaged on an LSM800 confocal microscope (Zeiss). Images are displayed as maximum intensity z-projections, bar = 10 μm. Data are representative of 3 independent experiments.
(TIF)

S7 Fig LBPA and NPC1 colocalize in Gefitinib treated cells. (A) HT1080 cells were treated with vehicle (DMSO, 0.1%), Gefitinib (5 μM), or NVP-ADW742 (2.5 μM) for 4 h. Cells were then fixed, permeabilized, and immunostained with rabbit anti-NPC1 and mouse anti-LBPA, followed by DY650-conjugated antiserum (Magenta) or AF555-conjugated antiserum (Green). Following immunostaining, cells were stained with Hoechst (Blue) and imaged on an LSM800 confocal microscope (Zeiss). Images are a cross-sectional view to visualize the Z coordinate axis, bar = 10 μm. (B) Pearson’s coefficient was determined per cell for each condition using Imaris (Bitplane) image analysis software. Data are representative of 3 independent experiments. * p < 0.05, ** p < 0.01, *** p < 0.001.
(TIF)

S8 Fig Kinetics of full-length EBOV GP VLP-induced Akt phosphorylation in Vero cells. Vero cells were serum-starved in HBSS for 1h followed by exposure to purified Mock supernatants or βlam VLPs harboring the full-length EBOV GP for 15, 30, or 60 min. Cells were washed, lysed, and immunoblotted for phosphorylated Akt (p-Akt S473). The membrane was then stripped and re-probed for total Akt. Data are representative of 2 independent experiments.
(TIF)

S9 Fig Viral particles harboring full-length EBOV GP and EBOV ΔM GP activate Akt in BMDMs. Murine bone-marrow derived macrophages were serum starved in serum-free RPMI for 1h followed by exposure to purified Mock, βlam EBOV ΔM GP, or βlam EBOV Full Length GP VLPs for 20 min. Cells were lysed and phosphorylated Akt (p-Akt—S473), total Akt (Akt), and GAPDH were detected by immunoblot. Data are representative of 3 independent experiments.
(TIF)

S10 Fig Phosphatidylserine is present on the viral envelope of both EBOV ΔM GP VLPs and Bald VLPs. GFP EBOVΔM GP VLPs and GFP Bald VLPs were stained with Annexin V-PE in ABB and analyzed using nanoscale flow cytometry (CytoFLEX S, Beckman Coulter). Unstained GFP EBOVΔM GP VLPs were run as a negative control (left). Data is representative of 3 independent experiments.
(TIF)
