# Supplementary material for: Ebola virus triggers receptor tyrosine kinase-dependent signaling to promote the delivery of viral particles to entry-conducive intracellular compartments
Source: PLoS Pathog. 2021 Jan 29;17(1):e1009275. doi: 10.1371/journal.ppat.1009275 (PMC7875390; doi:10.1371/journal.ppat.1009275)
Supplement: S1 Table — (DOCX) [file ppat.1009275.s002.docx]

| **Compound** | $\frac{\text{Mean EBOV}}{\text{Mean VSV}}$ | **p-value** ^a^ | **Targets** |
| --- | --- | --- | --- |
| Bosutinib (SKI-606) | 0.14744 | 0.00011 | Src |
| Cabozantinib malate (XL184) | 0.35110 | 0.00029 | VEGFR, TAM Receptor |
| IKK-16 (IKK Inhibitor VII) | 0.33990 | 0.00035 | IκB, IKK |
| Ehop-016 | 0.09569 | 0.00183 | Rho |
| LDK378 | 0.24043 | 0.00201 | ALK |
| PF-543 | 0.45908 | 0.00401 | S1P Receptor, SK1 |
| MK-2206 2HCl | 0.41949 | 0.00494 | Akt |
| G-749 | 0.12297 | 0.00590 | FLT3 |
| NVP-AEW541 | 0.23904 | 0.00635 | IGF-1R |
| NVP-ADW742 | 0.15585 | 0.00781 | IGF-1R, InsR |
| Crizotinib (PF-02341066) | 0.26316 | 0.01045 | ALK, c-Met |
| R428 (BGB324) | 0.39924 | 0.01072 | TAM Receptor |
| Cabozantinib (XL184, BMS-907351) | 0.40127 | 0.01322 | Tie-2, TAM Receptor, FLT3, VEGFR, c-Met, c-Kit |
| AZD3463 | 0.23228 | 0.01842 | ALK |
| Gefitinib (ZD1839) | 0.48905 | 0.01972 | EGFR |
| PHA-665752 | 0.49418 | 0.01987 | c-Met |
| Afatinib (BIBW2992) | 0.47394 | 0.02491 | EGFR, HER2 |
| Fingolimod (FTY720) HCl | 0.47949 | 0.03755 | S1P Receptor |
| SGI-1776 free base | 0.15245 | 0.03809 | Pim |
| SU11274 | 0.24481 | 0.04192 | c-Met |
| Pacritinib | 0.07734 | 0.04214 | FLT3, Jak |
| MGCD-265 | 0.41277 | 0.04714 | c-Met, Tie-2, VEGFR |

**Table 1. Hits from small molecule kinase screen for EBOV.**

Src, proto-oncogene tyrosine-protein kinase Src; VEGFR, vascular endothelial growth factor receptor; TAM Receptor, Tyro3, Axl, and Mer Receptors; ALK, anaplastic lymphoma kinase; S1P Receptor, sphingosine-1-phosphate receptor; SK1, sphingosine kinase 1; Akt, protein kinase B; FLT3, fms-like tyrosine kinase 3; IGF-1R, insulin-like growth factor 1 receptor; InsR, insulin receptor; c-Met, tyrosine protein kinase Met; Tie-2, angiopoietin-1 receptor; c-Kit, mast/stem cell growth factor receptor; EGFR, epidermal growth factor receptor; HER2, human epidermal growth factor receptor 2; Pim, proto-oncogene serine/threonine-protein kinase; Jak, janus kinase.

^a^ p-values were calculated using a two-tailed student’s t test
