# Supplementary material for: Ebola virus triggers receptor tyrosine kinase-dependent signaling to promote the delivery of viral particles to entry-conducive intracellular compartments
Source: PLoS Pathog. 2021 Jan 29;17(1):e1009275. doi: 10.1371/journal.ppat.1009275 (PMC7875390; doi:10.1371/journal.ppat.1009275)
Supplement: S2 Table — (DOCX) [file ppat.1009275.s003.docx]

| **Compound** | $\frac{\text{Mean EBOV}}{\text{Mean VSV}}$ | **p-value**^a^ | **Targets** |
| --- | --- | --- | --- |
| Tepotinib (EMD 1214063) | 0.33839 | 0.00006 | c-Met |
| ERK5-IN-1 | 0.14453 | 0.00027 | ERK |
| Bosutinib (SKI-606) | 0.33863 | 0.00061 | Src |
| IKK-16 (IKK Inhibitor VII) | 0.16121 | 0.00078 | IκB/IKK |
| AZD9291 | 0.31426 | 0.00118 | EGFR |
| NVP-AEW541 | 0.38270 | 0.00201 | IGF-1R |
| LDK378 | 0.30200 | 0.00268 | ALK |
| EHop-016 | 0.20754 | 0.00305 | Rho kinase |
| WH-4-023 | 0.33797 | 0.00329 | Src |
| NVP-ADW742 | 0.17288 | 0.00688 | IGF-1R, InsR |
| Torin 2 | 0.34467 | 0.00756 | mTOR, ATM/ATR |
| Ponatinib (AP24534 | 0.17750 | 0.00775 | Bcr-Abl, PDGFR, VEGFR, FGFR |
| G-749 | 0.38916 | 0.00843 | FLT3 |
| GSK1904529A | 0.08005 | 0.00939 | IGF-1R |
| R428 (BGB324) | 0.04676 | 0.01063 | TAM Receptors |
| PHA-665752 | 0.48744 | 0.01410 | c-Met |
| URMC-099 | 0.18381 | 0.01412 | MLK, LRRK, Abl, VEGFR |
| AZD3463 | 0.06306 | 0.01877 | ALK |
| Entrectinib (RXDX-101 | 0.42418 | 0.03475 | ALK, Trk receptor |
| WAY-600 | 0.47060 | 0.03693 | mTOR |
| WZ8040 | 0.31926 | 0.04530 | EGFR |
| Dacomitinib (PF299804, PF299) | 0.24645 | 0.04779 | EGFR |
| Dorsomorphin (Compound C | 0.05700 | 0.04837 | AMPK |

**Table 2. Hits from small molecule kinase screen for MARV.**

c-Met, tyrosine protein kinase Met; ERK, extracellular signal-regulated kinases; Src, proto-oncogene tyrosine-protein kinase Src; EGFR, epidermal growth factor receptor; IGF-1R, insulin-like growth factor 1 receptor; ALK, anaplastic lymphoma kinase; Rho; InsR, insulin receptor; mTOR, mammalian target of rapamycin; ATM, ataxia telangiectasia mutated; ATR, ATM and RAD3-related; PDGFR, platelet-derived growth factor receptor; VEGFR, vascular endothelial growth factor receptor; FGFR, fibroblast growth factor receptor; FLT3, fms-like tyrosine kinase 3; TAM Receptor, Tyro3, Axl, and Mer Receptors; MLK, mixed-lineage kinase; LRRK, leucine-rich repeat kinase; Trk, tropomyosin receptor kinase; AMPK, AMP-activated protein kinase.

^a^ p-values were calculated using a two-tailed student’s t test
