# Supplementary figures and images for: Ebola virus triggers receptor tyrosine kinase-dependent signaling to promote the delivery of viral particles to entry-conducive intracellular compartments
Source: PLoS Pathog. 2021 Jan 29;17(1):e1009275. doi: 10.1371/journal.ppat.1009275 (PMC7875390; doi:10.1371/journal.ppat.1009275)

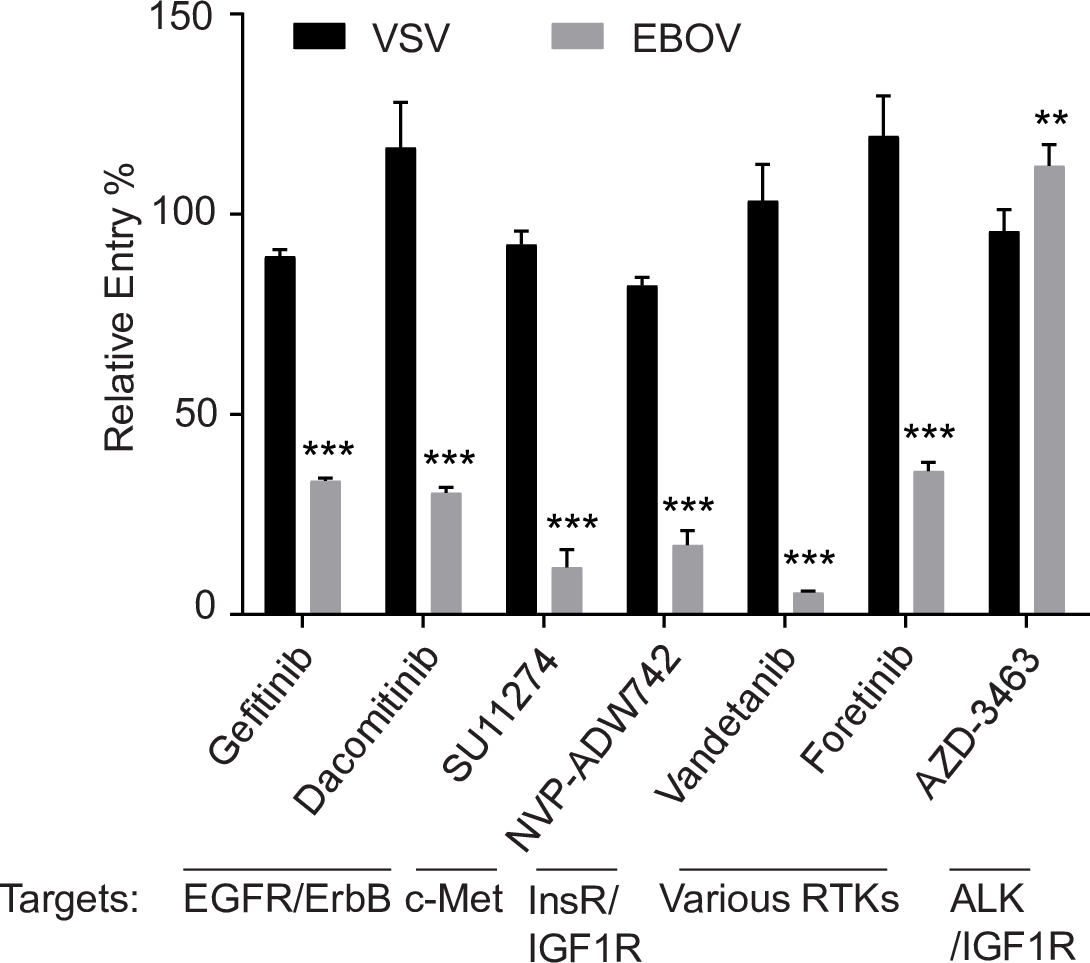

Supplement: S1 Fig — Vero cells were exposed to βlam VLPs harboring the EBOV GP or VSV G in the presence of vehicle (DMSO, 0.1%) or the indicated RTK inhibitor at 1 μM. Viral entry was detected via flow cytometry after loading cells with the βlam substrate, CCF2, and quantifying the percentage of cells with cleaved CCF2. Data are expressed as percentages of inhibitor treated cells relative to vehicle alone. Data are representative of 3 independent experiments. * p < 0.05, ** p < 0.01, *** p < 0.001. (TIF) [file ppat.1009275.s004.tif]

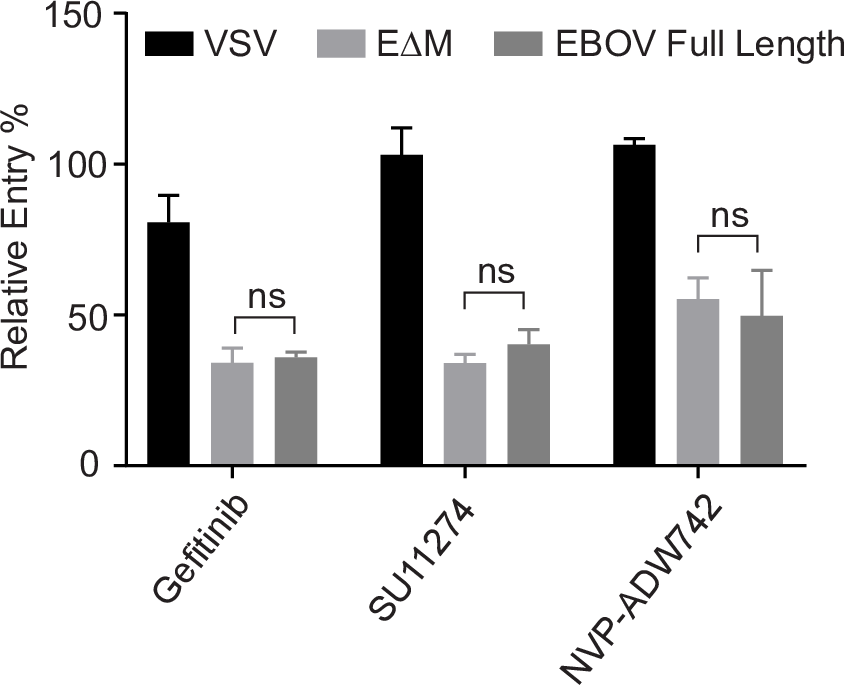

Supplement: S2 Fig — Vero cells were exposed to βlam VLPs harboring VSV-G, EBOV ΔM GP or the EBOV Full Length GP in the presence of vehicle (DMSO, 0.1%), Gefitinib (1 μM), SU11274 (500 nM), or NVP-ADW742 (500nM). Entry was detected via flow cytometry after loading cells with βlam substrate (CCF2) and measuring the percentage of cells with cleaved CCF2. Data are expressed as percentages of inhibitor treated cells relative to vehicle alone. Data are representative of 3 independent experiments. Students t-test was performed to compare % entry for EBOV ΔM GP and EBOV Full Length GP. (TIF) [file ppat.1009275.s005.tif]

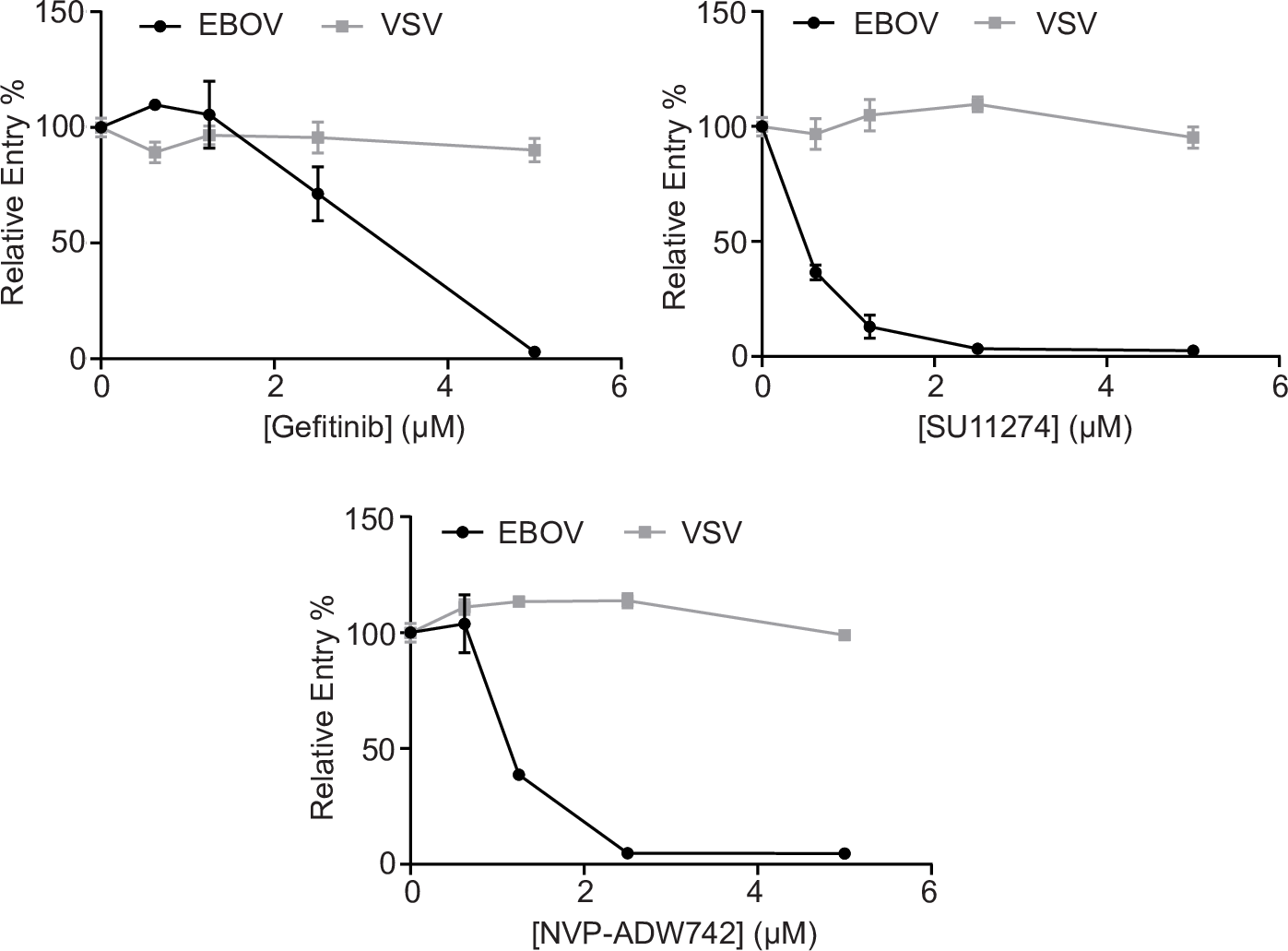

Supplement: S3 Fig — HT1080 were exposed to βlam VLPs harboring the EBOV GP or VSV G in the presence of vehicle (DMSO, 0.1%) or increasing concentrations of Gefitinib, SU11274, or NVP-ADW742. Entry was detected via flow cytometry after loading cells with βlam substrate (CCF2) and measuring the percentage of cells with cleaved CCF2. Data are expressed as percentages of inhibitor treated cells relative to vehicle alone. Data are representative of 3 independent experiments. (TIF) [file ppat.1009275.s006.tif]

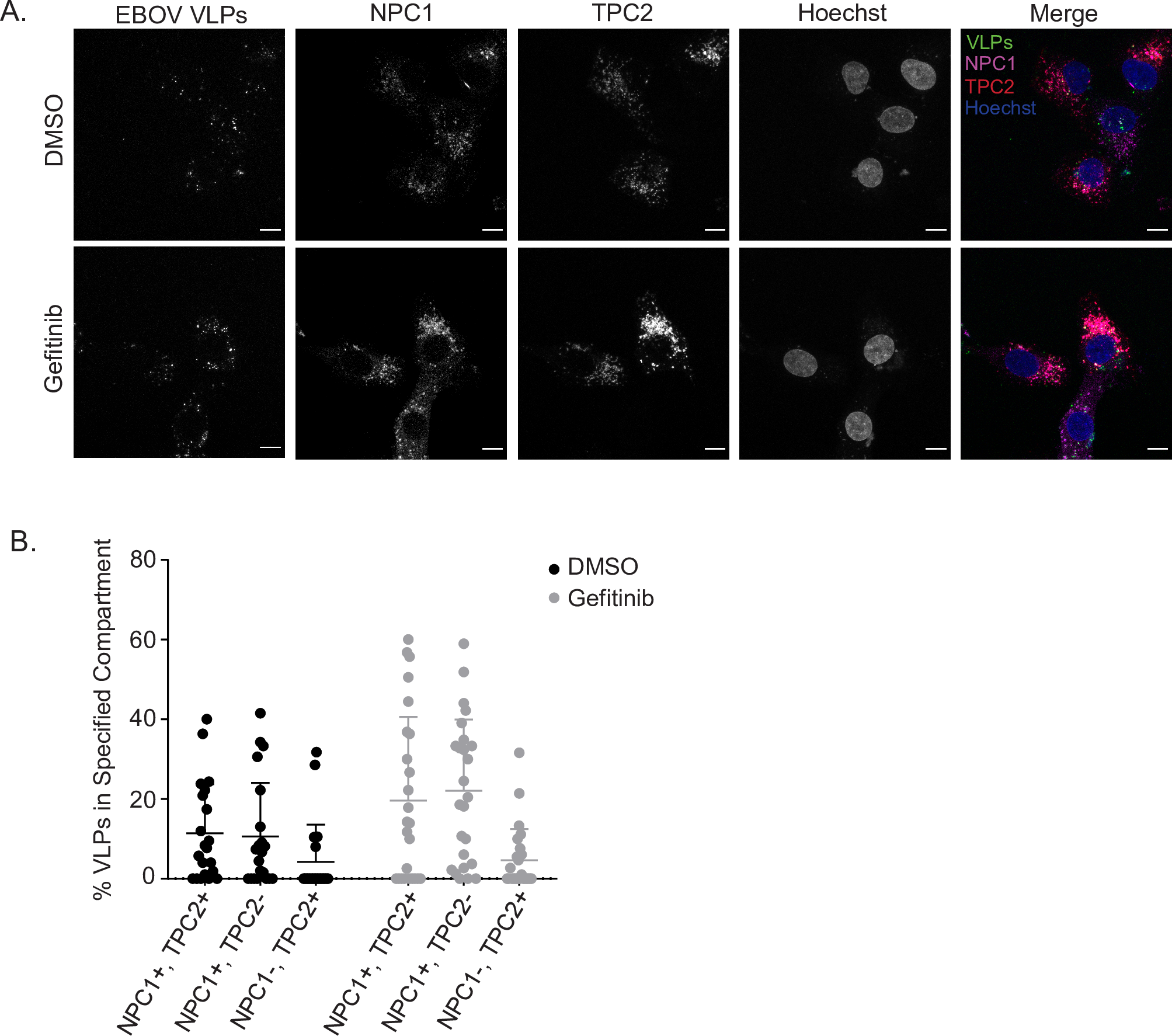

Supplement: S4 Fig — (A-B) HT1080 cells that were transfected with GFP-TPC2 (Red) and pre-treated with vehicle (DMSO, 0.1%) or Gefitinib (5 μM) were exposed to fluorescent VLPs (Green) harboring the fusion deficient ΔM GPF535R for 3 h. Cells were then fixed, permeabilized, immunostained with rabbit anti-NPC1 and DY650-conjugated antiserum (Magenta), and Hoechst (Blue). Cells were imaged on an LSM800 confocal microscope (Zeiss). Images in (A) are displayed as maximum intensity z-projections, bar = 10 μm. (B) Colocalization between VLPs and NPC1 and/or TPC2 were analyzed using Imaris software (Bitplane). Data are representative of 3 independent experiments. * p < 0.05, ** p < 0.01, *** p < 0.001. (TIF) [file ppat.1009275.s007.tif]

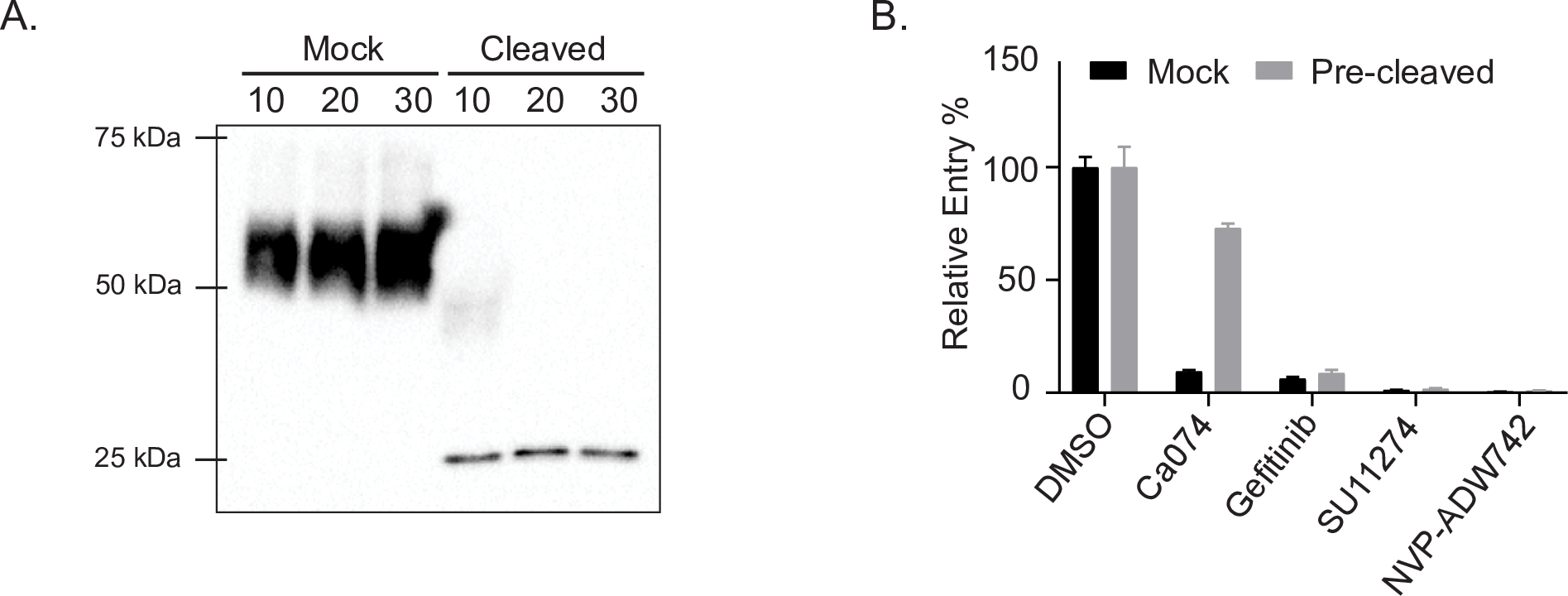

Supplement: S5 Fig — (A) βlam VLPs harboring the EBOV ΔM GP were incubated either with thermolysin (0.2 mg/mL) (Pre-cleaved) or PBS (Mock) for 10, 20, or 30 minutes prior to addition of phosphoramidon (500 μM). Lysates were prepared and immunoblotted for EBOV GP. (B) Pre-cleaved or mock virus that was incubated with thermolysin or PBS for 20 minutes was used to infect Vero cells treated with vehicle (DMSO, 0.1%), Ca074-Me (20 μM), Gefitinib (5 μM), SU11274 (2.5 μM), or NVP-ADW742 (2.5 μM). Entry was detected via flow cytometry after loading cells with βlam substrate (CCF2) and measuring the percentage of cells with cleaved CCF2. Data are expressed as percentages of inhibitor treated cells relative to vehicle alone. Data are representative of 3 independent experiments. (TIF) [file ppat.1009275.s008.tif]

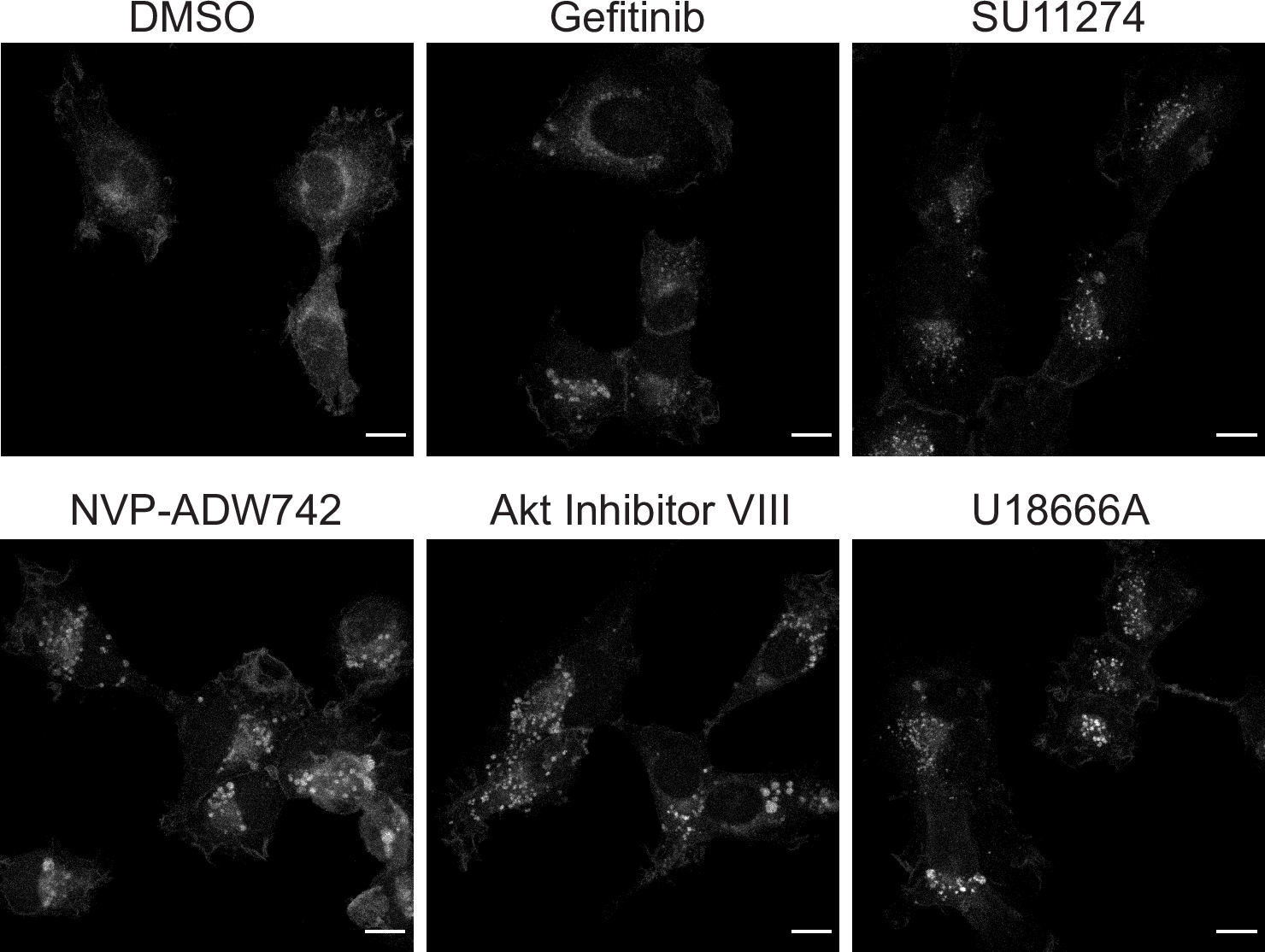

Supplement: S6 Fig — HT1080 cells were treated with vehicle (DMSO, 0.1%), Gefitinib (5 μM), SU11274 (2.5 μM), NVP-ADW742 (2.5 μM), Akt Inhibitor VIII (10 μM), or U18666A (5 μM) for 4 h. Cells were then fixed, stained with Filipin III, and imaged on an LSM800 confocal microscope (Zeiss). Images are displayed as maximum intensity z-projections, bar = 10 μm. Data are representative of 3 independent experiments. (TIF) [file ppat.1009275.s009.tif]

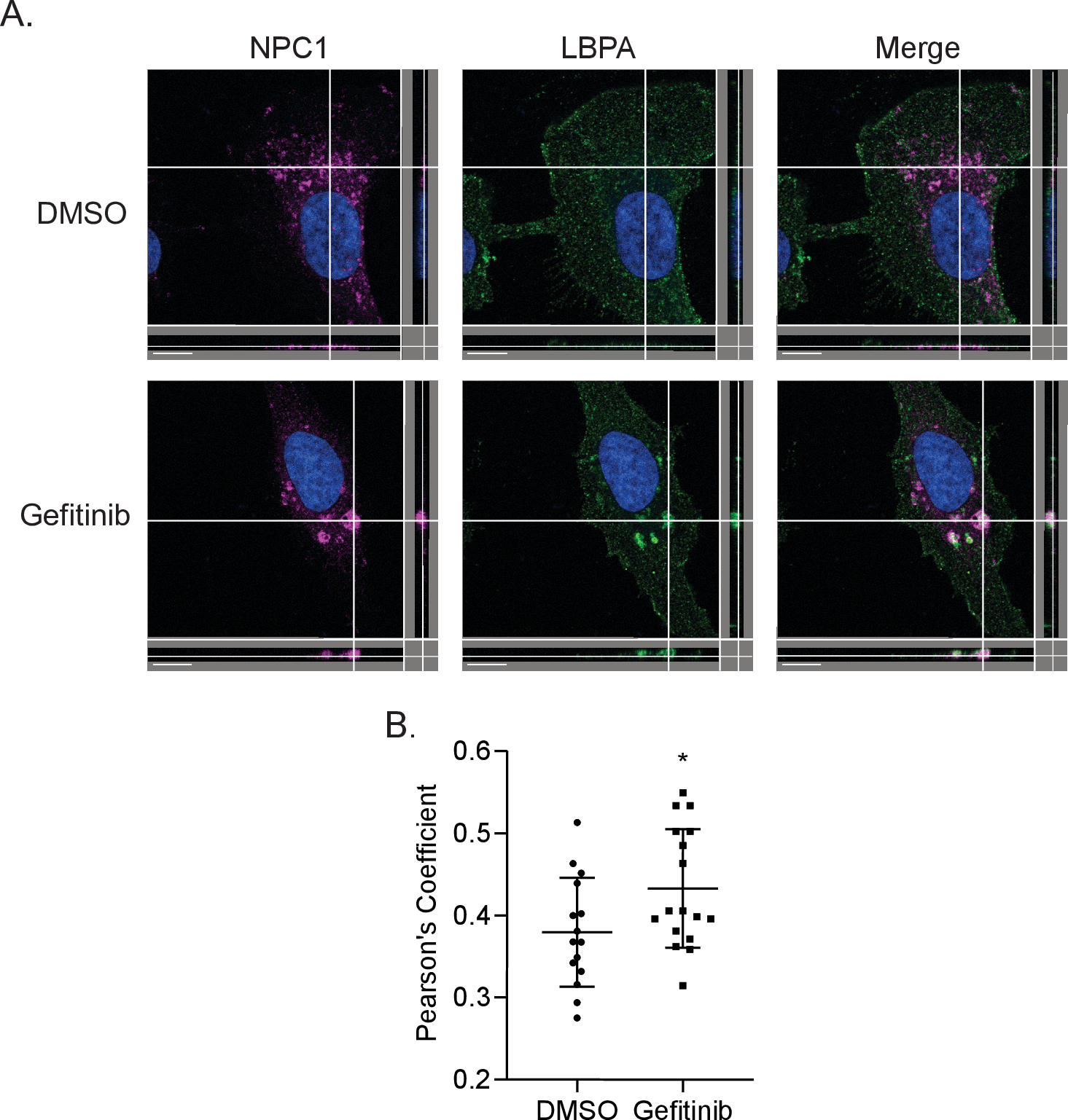

Supplement: S7 Fig — (A) HT1080 cells were treated with vehicle (DMSO, 0.1%), Gefitinib (5 μM), or NVP-ADW742 (2.5 μM) for 4 h. Cells were then fixed, permeabilized, and immunostained with rabbit anti-NPC1 and mouse anti-LBPA, followed by DY650-conjugated antiserum (Magenta) or AF555-conjugated antiserum (Green). Following immunostaining, cells were stained with Hoechst (Blue) and imaged on an LSM800 confocal microscope (Zeiss). Images are a cross-sectional view to visualize the Z coordinate axis, bar = 10 μm. (B) Pearson’s coefficient was determined per cell for each condition using Imaris (Bitplane) image analysis software. Data are representative of 3 independent experiments. * p < 0.05, ** p < 0.01, *** p < 0.001. (TIF) [file ppat.1009275.s010.tif]

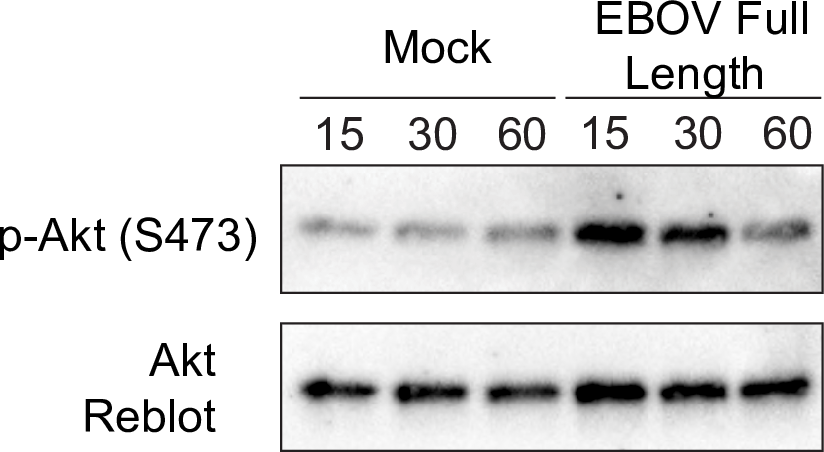

Supplement: S8 Fig — Vero cells were serum-starved in HBSS for 1h followed by exposure to purified Mock supernatants or βlam VLPs harboring the full-length EBOV GP for 15, 30, or 60 min. Cells were washed, lysed, and immunoblotted for phosphorylated Akt (p-Akt S473). The membrane was then stripped and re-probed for total Akt. Data are representative of 2 independent experiments. (TIF) [file ppat.1009275.s011.tif]

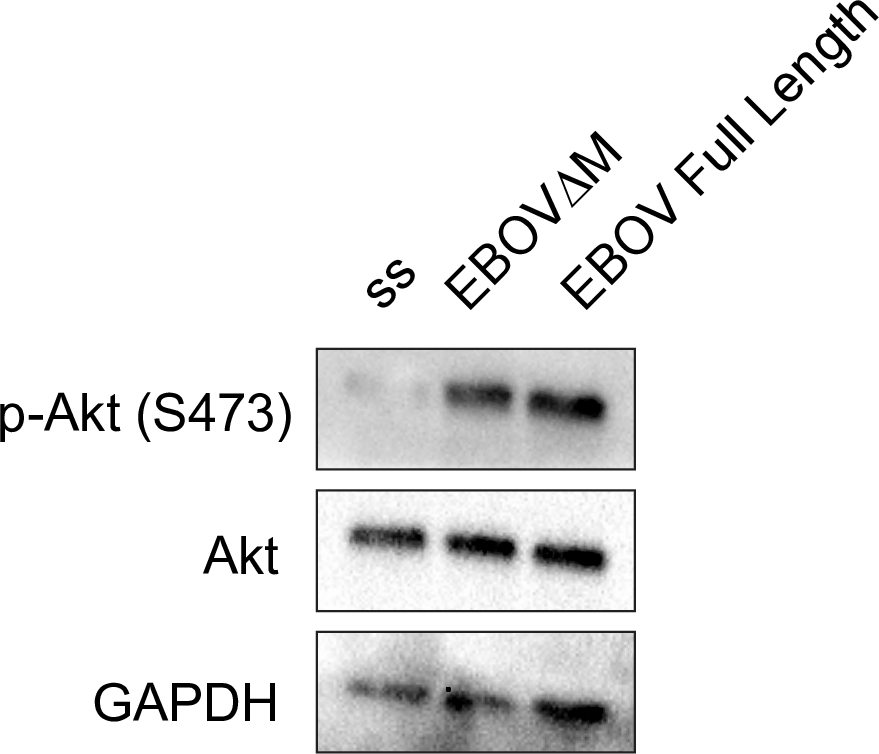

Supplement: S9 Fig — Murine bone-marrow derived macrophages were serum starved in serum-free RPMI for 1h followed by exposure to purified Mock, βlam EBOV ΔM GP, or βlam EBOV Full Length GP VLPs for 20 min. Cells were lysed and phosphorylated Akt (p-Akt—S473), total Akt (Akt), and GAPDH were detected by immunoblot. Data are representative of 3 independent experiments. (TIF) [file ppat.1009275.s012.tif]

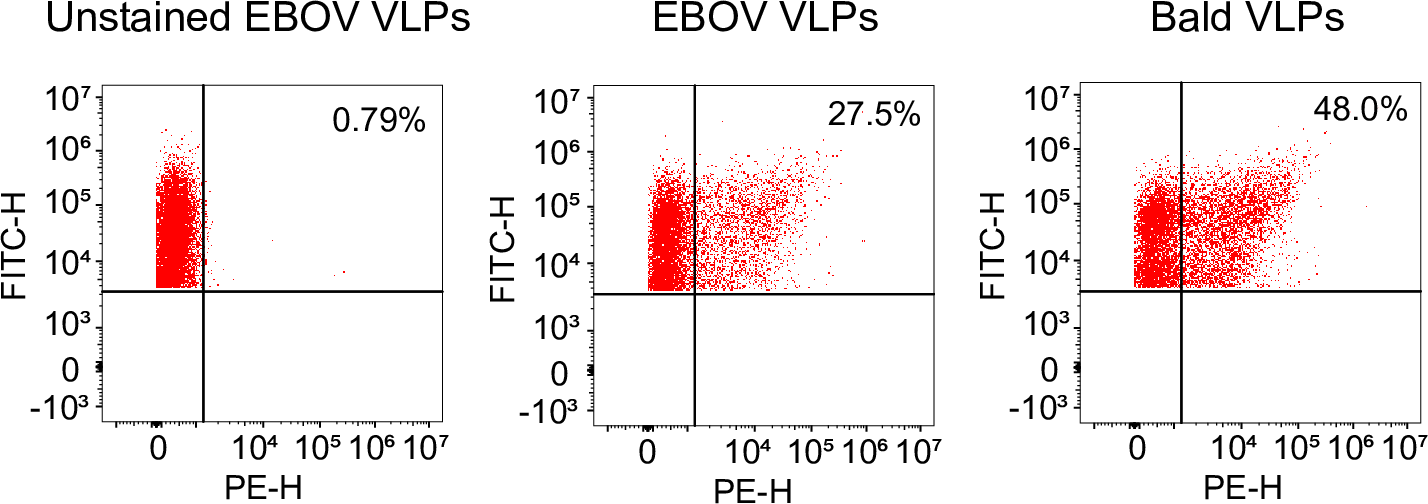

Supplement: S10 Fig — GFP EBOVΔM GP VLPs and GFP Bald VLPs were stained with Annexin V-PE in ABB and analyzed using nanoscale flow cytometry (CytoFLEX S, Beckman Coulter). Unstained GFP EBOVΔM GP VLPs were run as a negative control (left). Data is representative of 3 independent experiments. (TIF) [file ppat.1009275.s013.tif]
